# Serine Palmitoyltransferase Gene Silencing Prevents Ceramide Accumulation and Insulin Resistance in Muscles in Mice Fed a High-Fat Diet

**DOI:** 10.3390/cells11071123

**Published:** 2022-03-26

**Authors:** Monika Imierska, Piotr Zabielski, Kamila Roszczyc-Owsiejczuk, Emilia Sokołowska, Karolina Pogodzińska, Iwona Kojta, Agnieszka Błachnio-Zabielska

**Affiliations:** 1 Department of Hygiene, Epidemiology and Metabolic Disorders, Medical University of Bialystok, Mickiewicza 2c, 15-089 Bialystok, Poland; monika.imierska@umb.edu.pl (M.I.); kamila.roszczyc-owsiejczuk@umb.edu.pl (K.R.-O.); emiliasokolowska.umwb@gmail.com (E.S.); karolina.pogodzinska@umb.edu.pl (K.P.); paszkiewicziwona89@gmail.com (I.K.); 2 Department of Medical Biology, Medical University of Bialystok, Mickiewicza 2c, 15-089 Bialystok, Poland; piotr.zabielski@umb.edu.pl

**Keywords:** insulin resistance, ceramide, skeletal muscle lipid metabolism, gene silencing, electroporation

## Abstract

Skeletal muscles account for ~80% of insulin-stimulated glucose uptake and play a key role in lipid metabolism. Consumption of a high-fat diet (HFD) contributes to metabolic changes in muscles, including the development of insulin resistance. The studies carried out to date indicate that the accumulation of biologically active lipids, such as long-chain acyl-CoA, diacylglycerols and ceramides, play an important role in the development of insulin resistance in skeletal muscles. Unfortunately, it has not yet been clarified which of these lipid groups plays the dominant role in inducing these disorders. In order to explore this topic further, we locally silenced the gene encoding serine palmitoyltransferase (SPT) in the gastrocnemius muscle of animals with HFD-induced insulin resistance. This enzyme is primarily responsible for the first step of de novo ceramide biosynthesis. The obtained results confirm that the HFD induces the development of whole-body insulin resistance, which results in inhibition of the insulin pathway. This is associated with an increased level of biologically active lipids in the muscles. Our results also demonstrate that silencing the *SPT* gene with the shRNA plasmid reduces the accumulation of ceramides in gastrocnemius muscle, which, in turn, boosts the activity of the insulin signaling pathway. Furthermore, inhibition of ceramide synthesis does not significantly affect the content of other lipids, which suggests the leading role of ceramide in the lipid-related induction of skeletal muscle insulin resistance.

## 1. Introduction

Insulin resistance is a key link between obesity and type 2 diabetes. These disorders are related to a wide array of other pathophysiologic sequelae including dyslipidemia, hyperglycemia, hyperinsulinemia, hypertension, atherosclerosis and cardiovascular disease [1,2,3]. Altered lipid metabolism has the greatest effect on disturbance of insulin signaling in skeletal muscles which are responsible for 70–80% of the whole body insulin-stimulated glucose uptake, making them the most important site of insulin resistance [4].

Over the years, numerous hypotheses have been suggested to explain the mechanism by which increased lipid availability induces muscle insulin resistance [5,6,7,8]. Nowadays, fatty-acid-induced insulin resistance is most often attributed to intramuscular lipid accumulation. This view is strengthened by the fact that insulin resistance develops with the accumulation of fatty acid metabolites, especially long-chain fatty acyl-CoA (LCACoA), diacylglycerol (DAG) and ceramide (Cer), even in the absence of elevated levels of free fatty acids (FFA) in the plasma [9].

Ceramides belong to a group of lipid-derived molecules that consist of a sphingosine (SPH) base linked to a fatty acid moiety. They constitute structural elements of the membrane lipid bilayer and play a role as substantial signaling molecules that are implicated in a large number of cellular and physiological processes [10]. These lipid intermediates are accumulated in cells via two major routes: hydrolysis of the membrane phospholipid sphingomyelin, which is coordinated by the enzyme sphingomyelinase, or through de novo synthesis from long-chain acyl-CoA(LCA-CoAs), which involves a multi-step biosynthetic pathway [11]. The first rate-limiting step of de novo synthesis of ceramide is the condensation of acyl-CoA––usually palmitoyl-CoA––with serine, which is catalyzed by the enzyme serine palmitoyltransferase (SPT) [12], to form 3-ketosphinganine that is rapidly reduced to sphinganine (SPA) by the enzyme 3-ketosphinganine reductase. Next, SPA is acylated to form dihydroceramide by the action of dihydroceramide synthase. The last step of ceramide synthesis is the conversion of dihydroceramide to ceramide by insertion of a 4,5-trans-double bond into dihydroceramide. This reaction is catalyzed by the enzyme dihydroceramide desaturase [13].

As mentioned above, ceramides play important roles in cell biology, but their accumulation induces the development of metabolic dysfunction and insulin resistance in rodents and humans [14,15]. The connection between ceramide accumulation and insulin resistance is associated with protein kinase B (PKB/Akt) inhibition. PKB/Akt is a protein in the insulin pathway that directly participates in the translocation of GLUT4 to the plasma membrane, thus facilitating glucose uptake [16]. It has been demonstrated that ceramides are directly responsible for the activation of protein phosphatase 2A (PP2A) that triggers PKB/Akt dephosphorylation. Thus, ceramide inhibits the insulin pathway at this stage [17,18,19]. The second route by which ceramides are known to promote insulin resistance involves disruption of translocation GLUT4 to the membrane and reduced phosphorylation of PKB/Akt by a process dependent on the activation of atypical protein kinase C (aPKC) *λ*/*ζ* isoforms [20]. aPKCs, isoforms *λ*/*ζ* serve as actual molecular switches to activate GLUT4 translocation or glucose transport responses under the influence of insulin and certain other relevant stimuli in skeletal muscles and adipocytes [21].

Research results published to date have supported the hypothesis that ectopic lipid accumulation plays a fundamental role in the development of insulin resistance. However, there is an ongoing debate as to which group of lipid mediators plays the most important role here. Numerous published studies have confirmed the significant effect of intramuscular accumulation of ceramides. Studies with the SPT inhibitor myriocin or utilizing a *SPT* knockout in mice have shown that decreased ceramide levels protect against high-fat diet-induced insulin resistance [22]. However, the knockout animals or the use of inhibitors, reflect systemic changes that may result from altered metabolism in tissues other than muscle. The decrease in the level of ceramide in adipose tissue obtained in *SPT* knockout mice or by the use of myriocin, may alter the production and/or secretion of adipocytokines, which significantly affect skeletal muscle insulin sensitivity [23].

The aim of this study was to elucidate the role of ceramide accumulation in inducing muscle insulin resistance. To achieve this goal, the gene encoding SPT was locally silenced in the gastrocnemius muscle of HFD-fed animals.

## 2. Materials and Methods

### 2.1. Animals and Diets

The experiments were approved by the Local Ethics Committee for Animal Experiments (Olsztyn, Poland, approval number 43/2016). The study was performed using male C57BL/6 mice brought from the Jackson Laboratory (Bar Harbor, ME, USA). The animals were randomly divided into two groups, placed in standard conditions in a 12-h day/night cycle and provided with ad libitum access to food and water for 8 weeks, except when defined by the experimental protocol. The first group (*n* = 8), as the control, was fed a standard rodent diet containing 70% carbohydrates, 10% fat and 20% protein (% energy) (D12450J, Research Diets INC, New Brunswick, NJ, USA). The second group (*n* = 8) was fed a high-fat diet composed of 20% carbohydrates, 60% fat and 20% protein (% energy) (D12492, Research Diets INC, New Brunswick, NJ, USA).

### 2.2. Plasmid Preparation

Plasmids used in the experiment were obtained from bacterial stocks purchased from Dharmacon (currently: Horizon Discovery, Cambridge, UK). All plasmids produced by the bacteria encoded turbo green fluorescent protein (TurboGFP) gene as an exogenous expression control and shRNA targeted towards *Sptlc2*, a subunit of SPT––the enzyme responsible for de novo ceramide synthesis, or shRNA scrambled sequences. Bacterial cultures were prepared according to the manufacturer’s guidelines. Plasmids were isolated with the GeneJET Plasmid Maxiprep Kit (Thermo Scientific, Waltham, MA, USA), suspended in 150 mM PBS (pH = 7.2) and stored at −80 °C.

### 2.3. Plasmid Injection and Muscle Electroporation

**Figure 1:**
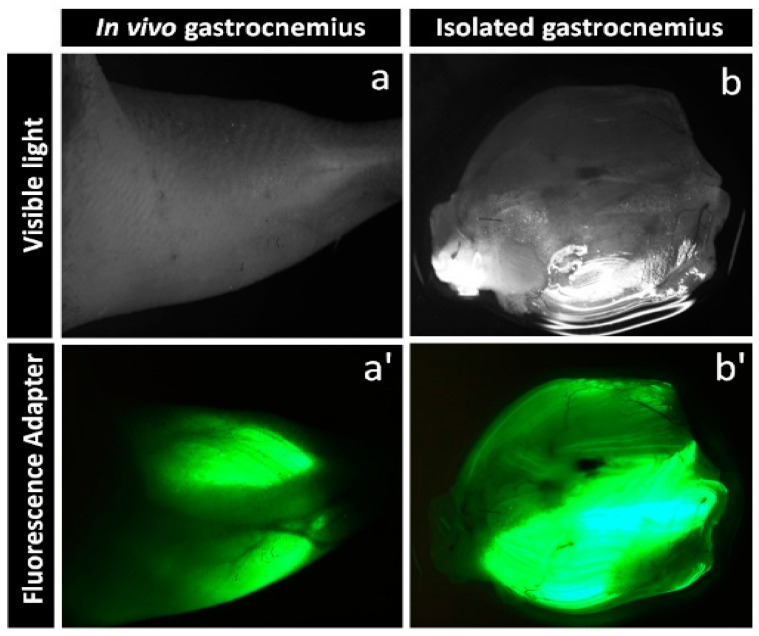
Green fluorescent protein (TurboGFP) in mouse muscle at 6 weeks after plasmid transfection and electroporation: (**a**) visible light photo of a mouse hindlimb; (**a’**) transcutaneous TurboGFP fluorescence of the gastrocnemius muscle; (**b**) visible light photo of isolated mouse gastrocnemius muscle; (**b’**) TurboGFP fluorescence of isolated gastrocnemius. Fluorescence stereomicroscopy performed with Nightsea SFA-RB-GO fluorescence adapter/DeltaPix Invenio 5SIII CMOS camera.

Gene silencing was performed two weeks after starting the allocated diets. In animals of all groups, the gastrocnemius muscle areas were shaved. Two hours prior to plasmid administration, 30 µL of hyaluronidase (0.4 U/µL hyaluronidase in sterile Tyrode) was injected in two equal doses from two sides of the muscle. The solution of 40 µg plasmid with a concentration of 2 µg/µL was slowly injected into the gastrocnemius muscle using a 27-gauge needle. Gastrocnemius muscles of both the hindlimbs in animals from the first group were treated with scrambled shRNA plasmid (control LFD). One hindlimb gastrocnemius muscle in mice from the fat-fed group was treated with shRNA plasmid mixture encoding three different sequences targeted towards *Sptlc2* (HFD_Sptlc2-shRNA_). The opposite hindlimb gastrocnemius muscle was treated with scrambled shRNA plasmids (HFD_+scrshRNA_) which activated shRNA-related mechanisms but preserved *Sptlc2* expression. After shRNA plasmid administration, the gastrocnemius muscles were electroporated with the use of a pair of stainless-steel electrode plates (1 cm^2^ area) and BTX ECM 830 Electroporation Generator (Holliston, MA, USA). During the electroporation procedure, eight electric pulses were applied at 200 ms intervals (175 volts/cm) [24]. Prior to all procedures, i.e., shaving, hyaluronidase/shRNA plasmid administration and electroporation, the animals were anesthetized in an induction chamber with ~2% isoflurane in oxygen with UNO BV rodent anesthesia system (UNO, Zevenaar, the Netherlands). GFP reporter gene expression in both legs was monitored transcutaneously once a week using a UV flashlight. The in-vivo GFP fluorescence at the time of euthanasia and after skeletal muscle excision was visualized with a stereomicroscope equipped with a Nightsea SFA-RB-GO add-on and DeltaPix Invenio 5SIII CMOS camera (Figure 1).

### 2.4. Insulin and Glucose Tolerance Tests

Six weeks after the commencement of the experimental diet in the animals, an oral glucose tolerance test (OGTT) was performed, followed by an insulin tolerance test (IPTT) one week later. For both procedures, the mice had been fasted for 6 h, and then baseline blood glucose concentrations measured from the tail vein by using glucometer AccuChek Aviva (Roche, Mannheim, Germany). Next, animals received glucose (in a dose of 2 g/kg) orally (OGTT) and insulin (dose of 0.75 U/kg) intraperitoneally (IPTG). Subsequent measurements of the glucose level were made at pre-determined intervals over 2–3 h (OGTT—15′, 30′, 45′, 60′, 90′, 120; IPTT—15′, 30′, 45′, 60′, 90′, 120; 180′). During OGTT at 15 and 60 min, blood was also collected for an insulin concentration assay with an ELISA insulin assay kit (Rat/Mouse Insulin Millipore, Merck KGaA, Darmstadt, Germany). The area under the glucose concentration curve was measured according to the trapezoidal rule.

### 2.5. HOMA-IR (Homeostatic Model Assessment for Insulin Resistance)

The HOMA-IR index value was calculated according to the formula [25]:

HOMA-IR = [fasting glucose (mg/dL) × fasting insulin (µIU/mL)]/2430(1)

### 2.6. Lipid Extraction and Analysis

#### 2.6.1. Sphingolipids

The content of sphingolipids was measured using a modified method according to Blachnio-Zabielska et al. [26]. The chromatographic lipid separation was conducted using an ultra-high performance liquid chromatography (Shimadzu Nexera X2 UHPLC, Shimadzu Corporation, Kyoto, Japan) in binary gradient with 1 mM ammonium formate and 0.1% formic acid in water (solvent A), and 2 mM ammonium formate and 0.1% formic acid in methanol (solvent B) at the flow rate of 0.4 mL/min. The analytical column was a reverse-phase Zorbax SB-C8 column 2.1 × 150 mm, 1.8 µm (Agilent Technologies, Santa Clara, CA, USA). The analysis was performed by means of a Sciex QTRAP 6500+ triple quadrupole mass spectrometer (AB Sciex Germany GmbH, Darmstadt, Germany) using a positive ion electrospray ionization (ESI) source (except for S1P, which was analyzed in the negative mode) with multiple reaction monitoring (MRM) against standard curves constructed for each compound. Sphingolipids were extracted from ~20 mg of pulverized skeletal muscle. Tissue samples were homogenized in 250 mM sucrose, 25 mM KCl, 50 mM Tris and 0.5 mM EDTA, pH 7.4. An internal standard solution was added to the homogenates (SPH-d7, SPA-d7, S1P-d7, C15:0-d7-Cer, C16:0-d7-Cer, C18:1-d7-Cer, C18:0-d7-Cer, 17C/20:0-Cer, C24:1-d7-Cer and C24-d7-Cer, Avanti Polar Lipids, Alabaster, AL, USA) and extraction mixture (isopropanol:water:ethyl acetate, 30:10:60; *v*/*v*/*v*). All samples were vortexed, sonicated and centrifuged. The extraction process was repeated.

The obtained supernatant was transferred to new vials and evaporated under a stream of nitrogen, and the dried samples were dissolved in solvent B.

#### 2.6.2. Diacylglycerols

The level of DAG was measured according to Blachnio-Zabielska et al. [27] by means of a triple quadrupole mass spectrometer (Sciex QTRAP 6500+ AB Sciex Germany GmbH, Darmstadt, Germany) and a positive ion electrospray ionization (ESI) source with multiple reaction monitoring (MRM) against the concentration standard curves prepared for each compound. Briefly, skeletal muscles were pulverized and homogenized, and internal standard mix (Deuterated DAG Mixture I and Mixture II, Avanti Polar Lipids, Alabaster, AL, USA) was added before the diacylglycerol extraction. The following DAG were quantified: C18:1/18:2, C16:0/18:2, C16:0/16:0, C16:0/18:1, C18:0/20:0, C18:0/18:1, C18:1/18:1, C18:0/18:2 and C16:0/18:0.

#### 2.6.3. Plasma FFA

Plasma FFA concentration was measured according to Persson et al. [28] by ultra-high performance liquid chromatography mass spectrometry (UHPLC/MS) against a six-point standard curve. Fatty acids were separated using a reverse-phase Zorbax SB-C18 column 2.1 × 150 mm, 1.8 μm (Agilent Technologies, Santa Clara, CA, USA) by means of a two-buffer system (buffer A: 80% acetonitrile and 0.5 mM ammonium acetate; buffer B: 99% acetonitrile and 1% 0.5 mM ammoniumacetate). An internal standard containing appropriate concentrations of C14:0-d27, C15:0, C16:0-d31, C17:0, C18:1-d9 and C18:0-d35 (Avanti Polar Lipids, Alabaster, AL, USA) was added to the plasma samples and then extractions were performed with freshly prepared Dole solution (isopropanol: heptane:1 M H_2_SO_4_, 40:10:1; *v*/*v*/*v*). The extracts were evaporated under a nitrogen stream and resuspended in buffer A for the LC/MS analysis.

#### 2.6.4. Triacylglycerols

The TAG content was measured by means of High Sensitivity Triglyceride Fluorometric Assay Kit (MAK264-1KT, Merck KGaA, Darmstadt, Germany) pursuant to the attached protocol.

#### 2.6.5. Malonyl-CoA and Long-Chain Acyl-CoA

Malonyl-CoA and LCA-CoA were extracted according to Minkler et al. [29] and their concentrations were measured according to Blachnio-Zabielska et al. [30]. Before extraction, the internal standard: C15:0-CoA, 16:0(d4)CoA, C17-CoA, C19:0-CoA, C21:0-CoA, C23:0-CoA and 24:0-(d4)-CoA (Avanti Polar Lipids, Alabaster, AL, USA) was added to all samples. The molecules were separated on a reversed-phase Agilent ZORBAX Extend-C18 column, 2.1 × 150 mm, using a binary gradient with ammonium hydroxide (NH_4_OH) in water and NH_4_OH in ACN. The concentration of LCA-CoA was quantified using multiple reaction monitoring (MRM) on a triple quadrupole mass spectrometer (Sciex QTRAP 6500+, AB Sciex Germany GmbH, Darmstadt, Germany) in positive electrospray ionization (ESI) mode against the concentration standard curves prepared for each compound (C14:0-CoA, C16:0-CoA, C16:1-CoA, C18:2-CoA, C18:1-CoA, C18:0-CoA, C20:0-CoA, C22:0-CoA, C24:1-CoA and C24:0-CoA, Avanti Polar Lipids, Alabaster, AL, USA).

#### 2.6.6. Acyl-Carnitines

Muscle acyl-carnitine content was measured based on a modified method of Giesbertz [31]. Pulverized gastrocnemius samples were homogenized and internal standard (C17-carnitine) was added. Next, the samples were extracted with the use of ice-cold methanol, centrifuged (10,000× *g*/4 °C/10 min), and the supernatants were dried under nitrogen in fresh tubes. After that, dried acyl-carnitine samples were derivatized to form butyl esters. In this step, the samples were shaken for 20 min at 60 °C in 100 µL n-butanol containing 5% *v*/*v* acetyl chloride. Then the samples were evaporated again, reconstituted in 100 µL methanol/water and transferred to glass vials for UHPLC/MS/MS analyses. Acyl-carnitines were quantified on a Sciex QTRAP 6500+ triple quadrupole mass spectrometer (AB Sciex Germany GmbH, Darmstadt, Germany) using positive ion electrospray ionization (ESI) with multiple reaction monitoring (MRM) against standard curves constructed for each compound. The chromatographic separation was performed with ultra-performance liquid chromatography (Shimadzu Nexera-X2 UHPLC). The analytical column was a reversed-phase Zorbax SB-C18 column 2.1 × 150 mm, 1.8 µm (Agilent Technologies, Santa Clara, CA, USA).

### 2.7. Western Blot Analysis

Gastrocnemius samples were homogenized in RIPA buffer (Merck KGaA, Darmstadt, Germany) with 0.5 mM tris(2-carboxyethyl)phosphine (TCEP, reducing agent, Merck KGaA, Darmstadt, Germany) containing protease and phosphatase inhibitors. Protein content in the homogenates was measured with the Pierce 660 nm protein assay kit (Thermo Fisher Scientific, Waltham, MA, USA). Bovine serum albumin (fatty acid-free) was used as a standard. Before protein separation by SDS-PAGE (AnykD Criterion TGX gels and Criterion Cell electrophoresis cell, BioRad, Hercules, CA, USA) and transferring it to PVDF membrane (BioRad Trans Blot SD semidry transfer cell with discontinuous buffer system: Tris/CAPS/15% methanol for anode and Tris/CAPS 0.1% SDS for cathode), the samples were denatured in Laemmli buffer. Finally, the membrane with the transferred proteins was incubated with the appropriate rabbit primary antibody. The following target proteins were quantified using rabbit primary antibodies: glucose transporter 4 (GLUT4) (Abcam, Cambridge, MA, USA), CD36 (Novus Biologicals, Centennial, CO, USA), FATP1 (Novus Biologicals, Centennial, CO, USA), FABPpm, creatinine O-palmitoyltransferase 1 (CPT1) (Abcam, Cambridge, MA, USA), Sptlc2, (Abcam, Cambridge, MA, USA), Akt, pAktSer473, IRS1, pIRS1 (Tyr632), pIRS1 (Ser1101) (Cell Signalling Technology, Danvers, MA, USA) and glyceraldehyde 3-phosphate dehydrogenase (GAPDH) (Abcam, Cambridge, MA, USA). Binding of the primary antibody was detected after incubation with HRP-conjugated secondary antibody, Clarity^™^ Western ECL chemiluminescent substrate (Bio-Rad), and visualized using the Bio-Rad ChemiDoc XRS+ imaging system. Band intensities were quantified with the Image Lab 6.1. (Bio-Rad Software). Values were normalized to GAPDH referent protein expression measured from parallel runs and expressed as fold changes over control group values. All chemical substances as well as the equipment used for immunoblotting were purchased from Bio-Rad.

### 2.8. RT-PCR

Total RNA was isolated from gastrocnemius muscles using the mirVana Isolation Kit (ThermoScientific, Waltham, MA, USA) according to the manufacturer’s protocol. The RNA was reverse-transcribed into cDNA using the Transcriptor First Strand cDNA Synthesis Kit (Roche, Mannheim, Germany). Real-time PCR was performed with RealTime Ready Custom Assays for *Sptlc2* and *Gapdh* as referent genes using a LightCycler480 system (Roche, Mannheim, Germany). The results were normalized to *Gapdh* expression measured in each sample.

### 2.9. Statistical Analysis

Results were expressed as medians and interquartile ranges (25th–75th percentiles, IQR). The significance of change was estimated with the Mann–Whitney U non-parametric test. Normality of the data was not assumed. The significance threshold was set at *p* < 0.05. Statistical analysis was performed using Prism 9.3.1. (GraphPad Software).

## 3. Results

### 3.1. Gene Silencing

**Figure 2:**
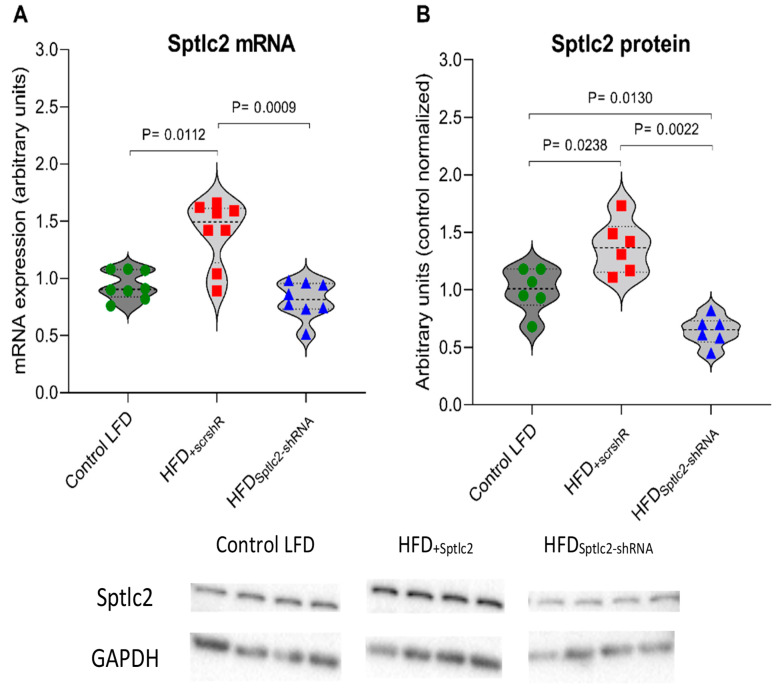
(**A**,**B**) Expression of skeletal muscle *Sptlc2* at the (**A**) mRNA (*n* = 8) and (**B**) protein level (*n* = 6). Significance by Mann–Whitney U test. The figures show the median and interquartile range. The observed molecular weights of indicated proteins are different from the theoretical values, as stated by the antibody manufacturer. Green circles—values for individual Control LTD gastrocnemius muscles; Red squares—values for individual HFD_+scrshRNA_ gastrocnemius muscles; Blue triangles—values for individual HFD_Sptlc2-shRNA_ gastrocnemius muscles.

The first evidence of proper functioning of intramuscularly administered plasmids was Green Fluorescent Protein (GFP) expression. It was observed transcutaneously just one day after electroporation and it was noticeable until tissue harvesting (Figure 1). The silencing of the gene responsible for the expression of *Sptlc2* was determined by an analysis of the levels of mRNA and Sptlc2 protein. Both, mRNA and the protein level of Sptlc2 significantly increased in the gastrocnemius muscle of HFD-fed animals (HFD_+scrshRNA_) compared with control LFD animals and significantly decreased in the HFD_Sptlc2-shRNA_ muscle compared with HFD_+scrshRNA_ (Figure 2).

### 3.2. Plasma Free Fatty Acid (FFA) Concentration

High-fat diet feeding resulted in a significant increase in total plasma free-fatty acid concentrations. The total level of FFA in the HFD-fed animals increased significantly and was almost twice as high as in the control LFD group (*p* < 0.05). More than a two-fold increase in concentration was observed for 9 out of 13 fatty acids tested: C14:0, C18:0, C18:1, C18:2, C20:4, C20:0, C22:0, C24:1 and C24:0 (*p* < 0.05) (Table 1).

### 3.3. Fatty Acid Transporters: CD36, FATP1 and FABPpm

**Figure 3:**
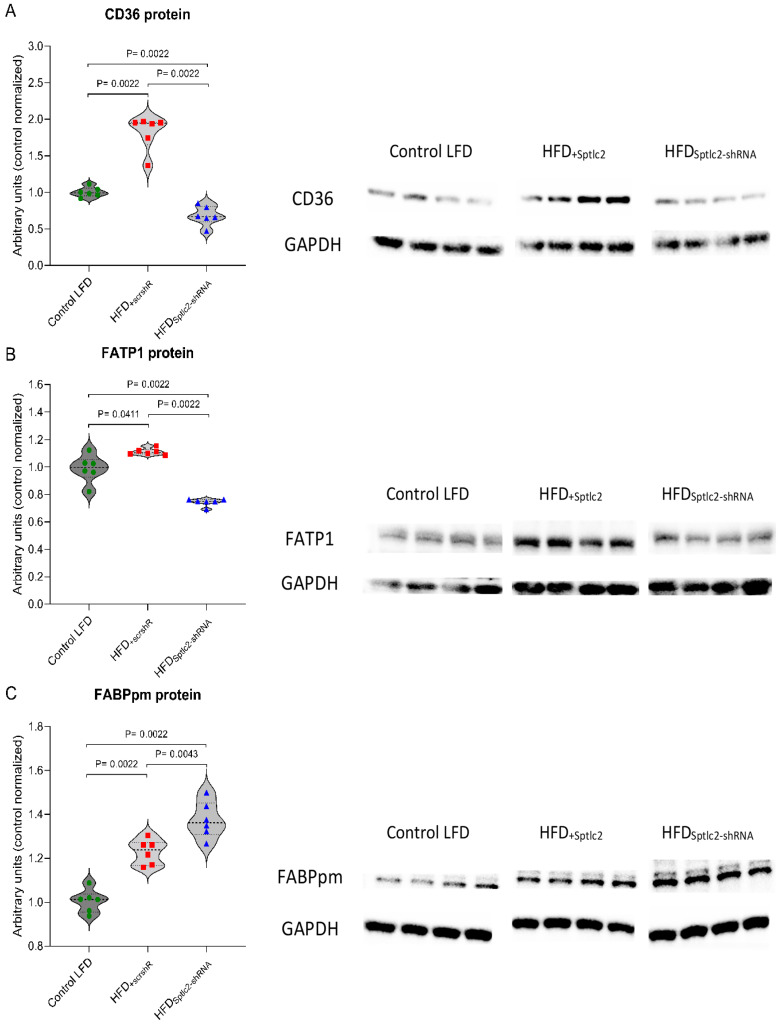
(**A**–**C**) The impact of skeletal muscle *Sptlc2* silencing on the protein expression of fatty acid transporters in the gastrocnemius muscle of high-fat diet mice: (**A**) protein expression of CD36; (**B**) protein expression of FATP1; (**C**) protein expression of FABPpm. Significance by Mann–Whitney U test. The figures show the median and interquartile range (*n* = 6). The observed molecular weights of the indicated proteins are different from the theoretical values, as stated by the antibody manufacturer. Green circles—values for individual Control LTD gastrocnemius muscles; Red squares—values for individual HFD_+scrshRNA_ gastrocnemius muscles; Blue triangles—values for individual HFD_Sptlc2-shRNA_ gastrocnemius muscles.

The levels of CD36, FATP1 and FABPpm were significantly increased in HFD_+scrshRNA_ skeletal muscles compared with control LFD muscles (*p* < 0.05). *Sptlc2* gene silencing in the muscles of HFD-fed animals significantly reduced the levels of CD36 and FATP1 compared with the HFD_+scrshRNA_ muscle (*p* < 0.05) (Figure 3A,B). The content of these transporters in the silenced gastrocnemius was also significantly lower than in control LFD muscles (*p* < 0.05). *Sptlc2* silencing in the gastrocnemius muscle of high-fat diet mice increased the level of FABPpm to significantly higher than in the HFD_+scrshRNA_ (Figure 3C).

### 3.4. Ceramide Content

**Figure 4:**
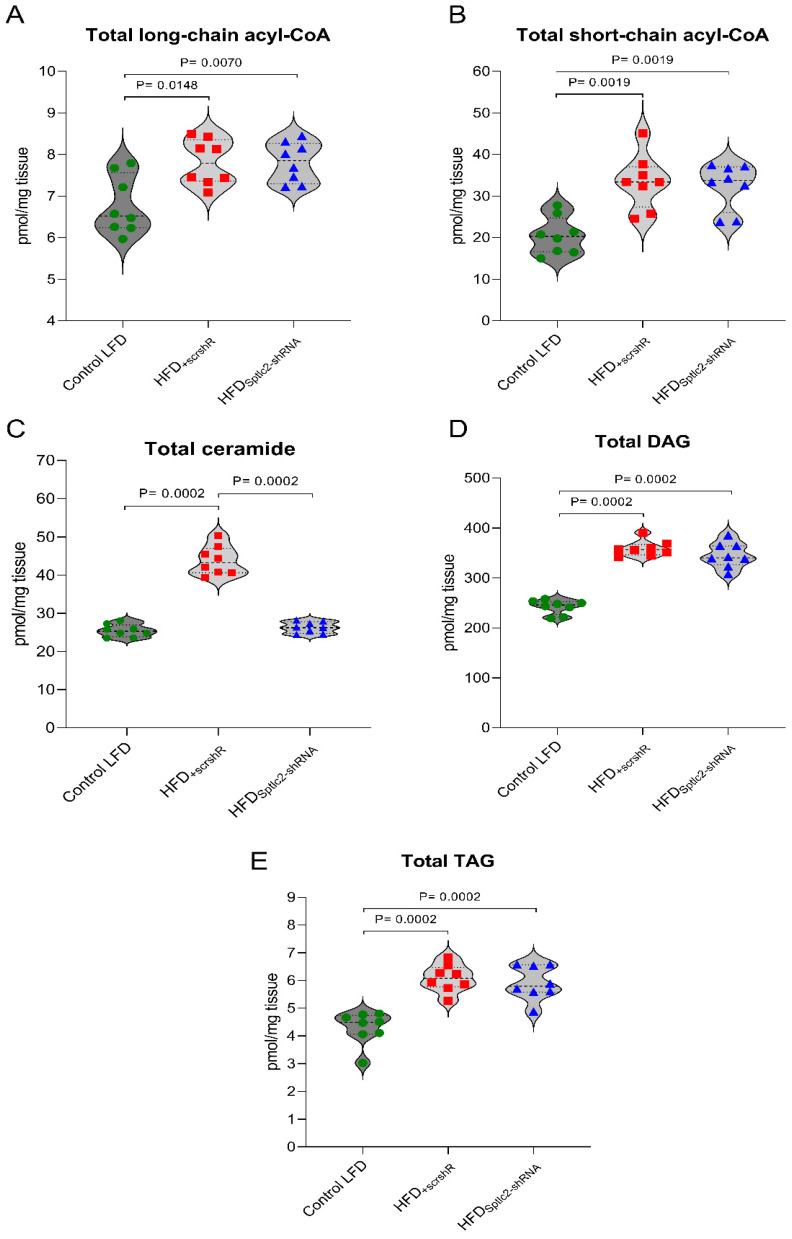
(**A**–**E**) The impact of skeletal muscle *Sptlc2* silencing on the content of bioactive lipids: (**A**) the content of long-chain acyl-CoA; (**B**) the content of short-chain acyl-CoA; (**C**) the content of ceramide; (**D**) the content of diacylglycerols; (**E**) the content of triacylglycerol. Significance by Mann–Whitney U test. The figures show the median and interquartile range (*n* = 8). Green circles—values for individual Control LTD gastrocnemius muscles; Red squares—values for individual HFD_+scrshRNA_ gastrocnemius muscles; Blue triangles—values for individual HFD_Sptlc2-shRNA_ gastrocnemius muscles.

The total content of ceramide increased significantly in the HFD_+scrshRNA_ gastrocnemius muscle compared with the ceramide content in the muscle of the control LFD group (*p* < 0.05) (Figure 4C). Feeding with a high-fat diet increased the level of all analyzed ceramides except C14-Cer. The high-fat diet did not notably affect C14:0-Cer content (Table 2). *Sptlc2* silencing in the gastrocnemius muscle of high-fat diet mice significantly decreased the content of all the measured sphingolipids to values observed in the control LFD group (*p* < 0.05) (Table 2).

High-fat diet feeding resulted in a considerable increase in sphinganine (Table 3), sphingosine (Table 3) and sphingosine-1-phosphate (Table 3) compared with gastrocnemius muscle of the control LFD group. *Sptlc2* gene silencing in the gastrocnemius muscle of HFD-fed animals resulted in a significant decrease in the content of these compounds (*p* < 0.05) to the level observed in the LFD control group.

### 3.5. Skeletal DAG and TAG Content

The total content of DAG and TAG significantly increased in HFD_+scrshRNA_ muscles compared with the control LFD group (*p* < 0.05). *Sptlc2* silencing did not trigger changes in either total DAG or TAG concentration (Figure 4D,E). Analyzing individual species of DAGs, we observed that C16:0/18:0, C16:0/18:2 and C18:0/20:0 concentrations demonstrated the same tendency as total DAG. Their level increased in the HFD_+scrshRNA_ group but was not affected by *Sptlc2* silencing (*p* < 0.05). The concentration of C16:0/16:0 and C18:0/18:2 significantly increased in the muscles of the HFD_+scrshRNA_ group animals compared with the control LFD group (*p* < 0.05). *Sptlc2* silencing resulted in an even greater, statistically significant increase compared with both control LFD and HFD_+scrshRNA_ groups (*p* < 0.05). The level of C18:0/18:1 did not rise in the HFD_+scrshRNA_ muscles. *Sptlc2* silencing significantly increased the level of this DAG compared with both control LFD and HFD_+scrshRNA_ muscle (*p* < 0.05). In HFD_+scrshRNA_ and HFD_Sptlc2-shRNA_ muscle, 18:1/18:1 level was similar to that in the control LFD group (Table 4).

### 3.6. Skeletal Muscle Short- and Long-Chain Acyl-CoA

The contents of both long- and short-chain acyl-CoA increased significantly in HFD_+scrshRNA_ skeletal muscles (*p* < 0.05). The highest increase was observed for C14:0-CoA, C16:0-CoA, C18:0-CoA and C22:0-CoA (*p* < 0.05) (Table 5). *Sptlc2* silencing did not affect its content, which remained at a similar level to that in the HFD_+scrshRNA_ muscles (Figure 4A,B).

### 3.7. Skeletal Muscle Long-Chain Acyl-Carnitine and CPT1B Content

**Figure 5:**
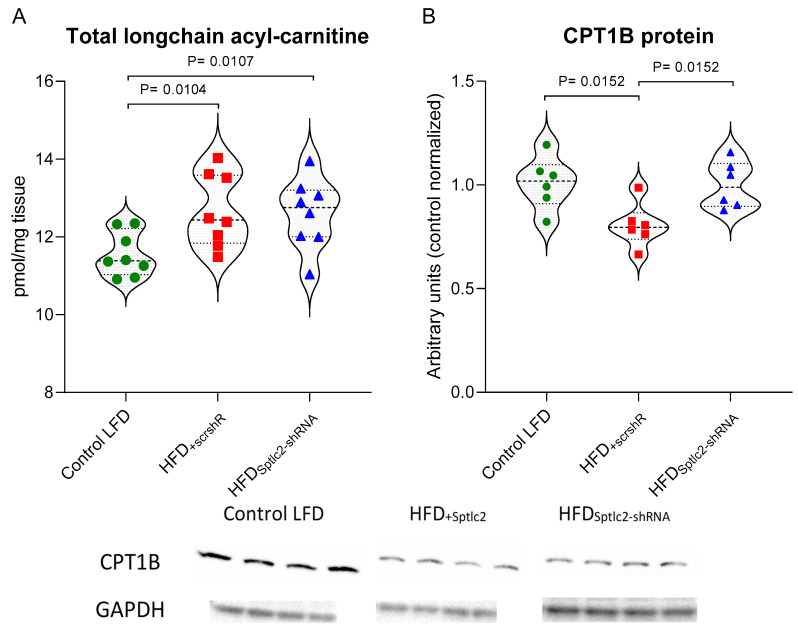
(**A**,**B**) The impact of skeletal muscle *Sptlc2* silencing on the content of acyl-carnitines and the protein expression of CPT1B: (**A**) the content of long-chain acyl-carnitines (*n* = 8); (**B**) protein expression of CPT1B (*n* = 6). Significance by Mann–Whitney U test. The figures show the median and interquartile range. The observed molecular weights of the indicated proteins are different from the theoretical values, as stated by the antibody manufacturer. Green circles—values for individual Control LTD gastrocnemius muscles; Red squares—values for individual HFD_+scrshRNA_ gastrocnemius muscles; Blue triangles—values for individual HFD_Sptlc2-shRNA_ gastrocnemius muscles.

The content of long-chain acyl-carnitines was significantly higher in HFD_+scrshRNA_ muscles than in the muscles of LFD-fed control animals (*p* < 0.05). The highest increase was observed for C18:0-carnitine. In the case of C14:0-carnitine, a significant decline was observed in the HFD_+scrshRNA_ and HFD_Sptlc2-shRNA_ muscles compared with the LFD control (Table 6). *Sptlc2* silencing did not affect its content, which remained at a similar level to that observed in the HFD_+scrshRNA_ muscle (Figure 5A).

The content of CPT1B significantly decreased in the HFD_+scrshRNA_ muscles compared with the control LFD. *Sptlc2* silencing increased CPT1B content in skeletal muscles to a level significantly higher than in the HFD_+scrshRNA_ muscles (*p* < 0.05) and values not significantly different from those observed in the control LFD group (Figure 5B).

### 3.8. Insulin Sensitivity

**Figure 6:**
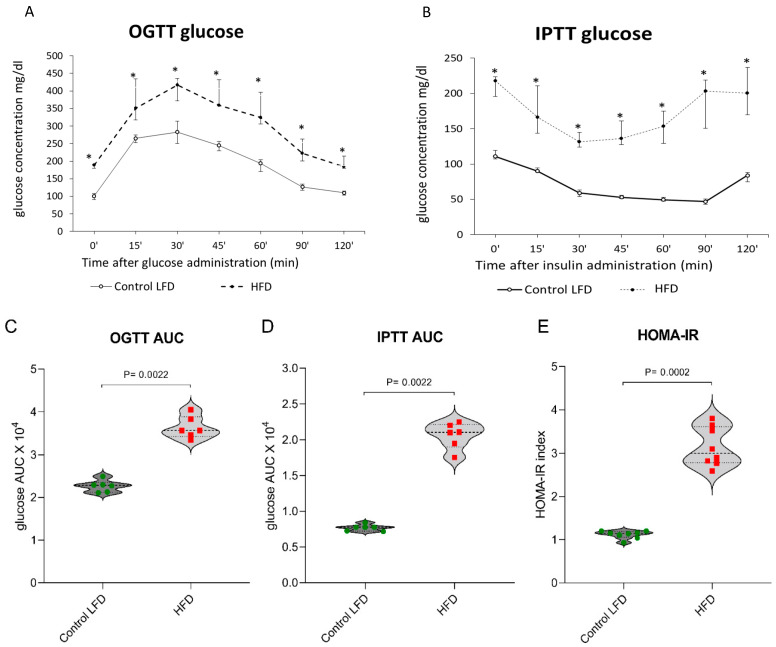
(**A**–**E**) The impact of high-fat diet consumption on plasma glucose concentration: (**A**) plasma glucose profile during the oral glucose tolerance test (OGTT); (**B**) plasma glucose profile during the intraperitoneal insulin tolerance test (IPTT); (**C**) area under the plasma glucose curve for OGTT (IPTT); (**D**) area under the plasma glucose curve for IPTT; (**E**) HOMA-IR value. Significance by Mann–Whitney U test. The figures show the median and interquartile range (*n* = 6 per group). Green circles—values for individual Control LTD gastrocnemius muscles; Red squares—values for individual HFD_+scrshRNA_ gastrocnemius muscles. * *p* < 0.05.

HFD-fed mice developed insulin resistance, as evidenced by their elevated fasting glucose and insulin levels. Impaired glucose tolerance, decreased insulin responsiveness and an increase in the HOMA-IR values were also observed compared with the control LFD group (Figure 6A–E).

### 3.9. Insulin Signaling Cascade

**Figure 7:**
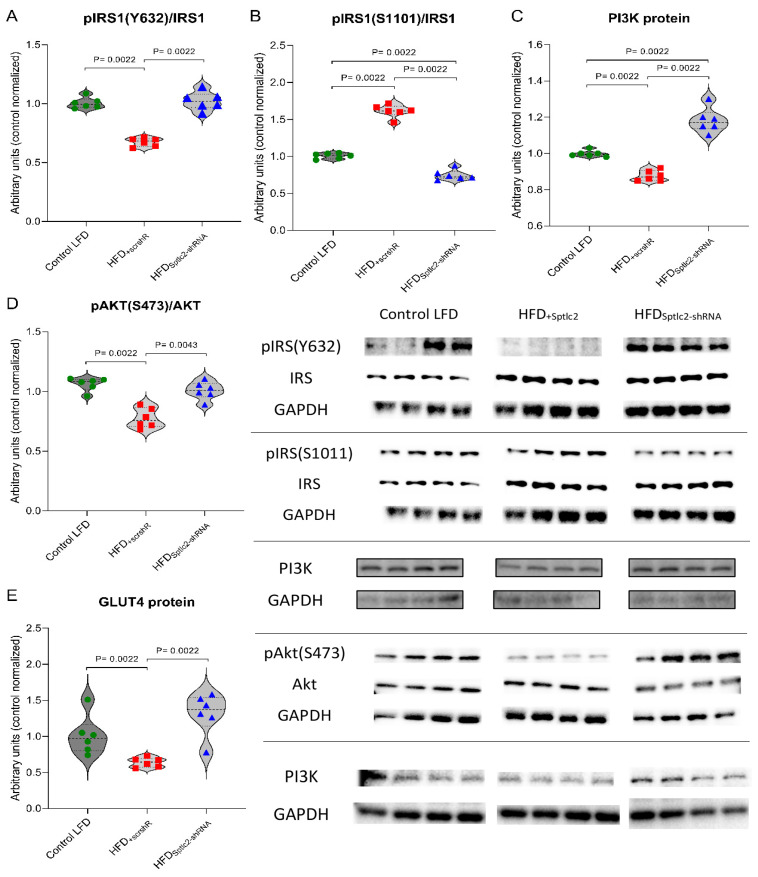
(**A**–**E**) Effect of *Sptlc2* silencing on activation of insulin signaling pathways in the mouse gastrocnemius muscle: (**A**) insulin receptor substrate 1 (IRS1) tyrosine phosphorylation (pIRS1 Y632); (**B**) IRS1 serine phosphorylation (pIRS1 S1101); (**C**) protein expression of phosphoinositide 3-kinase (PI3K); (**D**) serine phosphorylation of protein kinase B/Akt (pAKT S473); (**E**) protein expression of glucose transporter 4 (GLUT4). Significance by Mann–Whitney U test. The figures show the median and interquartile range (*n* = 6 per group). The observed molecular weights of the indicated proteins are different to the theoretical values, as stated by the antibody manufacturer. Green circles—values for individual Control LTD gastrocnemius muscles; Red squares—values for individual HFD_+scrshRNA_ gastrocnemius muscles; Blue triangles—values for individual HFD_Sptlc2-shRNA_ gastrocnemius muscles.

The level of tyrosine phosphorylation of the insulin receptor substrate 1 (IRS1) significantly decreased in the HFD_+scrshRNA_ muscles compared with the control LFD group (*p* < 0.05). *Sptlc2* silencing increased this value to the level observed in the muscles of the control LFD group (Figure 7A). The degree of IRS1 serine residue (S1101) phosphorylation, was significantly higher in HFD_+scrshRNA_ muscles compared with the muscles of control animals fed LFD (*p* < 0.05). In HFD_Sptlc2-shRNA_ muscles, the degree of serine phosphorylation decreased below the value observed in both HFD_+scrshRNA_ and LFD muscles (*p* < 0.05) (Figure 7B). It should be emphasized that phosphorylation of tyrosine residues in IRS1 activates insulin signal transduction, while phosphorylation of serine residues in IRS1 inhibits the activity of the insulin pathway [32].

The protein content of PI3K decreased in HFD_+scrshRNA_ muscles compared with the control LFD group (*p* < 0.05). A significant increase in the amount of this protein was observed in the HFD_Sptlc2-shRNA_ muscles in comparison with both the control LFD and HFD_+scrshRNA_ muscles (*p* < 0.05) (Figure 7C).

The level of serine phosphorylation of Akt protein dropped considerably in HFD_+scrshRNA_ muscles compared with the control LFD muscles (*p* < 0.05), and increased in HFD_Sptlc2-shRNA_ muscles to values not significantly different from those observed in the control LFD mice (Figure 7D).

The content of the GLUT4 protein significantly decreased in the HFD_+scrshRNA_ group compared with the control LFD group (*p* < 0.05), and was normalized in HFD_Sptlc2-shRNA_ skeletal muscles, compared with the HFD_+scrshRNA_ mice without gene silencing (*p* < 0.05), to values not significantly different from those observed in the control LFD group (Figure 7E).

## 4. Discussion

Numerous studies suggest that exposing the muscle tissue to saturated free fatty acids triggers the accumulation of ceramide that has been shown to inhibit the early steps of insulin signaling [33,34,35]. The goal of this research was to determine the extent to which biologically active lipid ceramide, leads to the development of disorders associated with skeletal muscle insulin resistance, under the conditions of increased supply of fatty acids.

To achieve the intended goal, the expression of gene encoding serine palmitoyltransferase (Sptlc2), a key enzyme involved in de novo ceramide synthesis, was locally silenced in the gastrocnemius muscle of HFD-fed mice by electroporation-mediated, shRNA plasmid-based transfection. To isolate the effects of *Sptlc2* silencing, one mouse hindlimb was silenced with shRNA plasmids (HFD_Sptlc2-shRNA_ muscle), while the muscle of the other limb was transfected with scrambled shRNA plasmids (HFD_+scrshRNA_ muscle).

As expected, increasing dietary fat intake triggered insulin resistance at both the systemic and muscular level. Decreased insulin sensitivity was manifested by increased fasting glucose and insulin levels, impaired glucose tolerance, decreased insulin sensitivity, and increased HOMA-IR. The same effects of consuming a high-fat diet have also been reported in other studies [23,36,37,38,39,40,41]. In addition, it has also been confirmed that the HFD diet leads to an increase in total free fatty acids in plasma. Increased plasma FFA levels associated with the consumption of a high-fat diet have also been observed in other human and rodent studies [42,43,44,45].

In the studies conducted here, we also found that the elevated plasma FFA concentration was accompanied by an increase in the level of the proteins CD36, FATP1 and FABPpm involved in the transport of fatty acids in skeletal muscles. The same results were obtained by Yun et al. [46] and other researchers [47,48,49]. They confirmed the effect of a HFD on an increase of fatty acid transporter expression. In our study, silencing of *Sptlc2* gene resulted in a significant decrease in the content of CD36 and FATP1 in high-fat diet-fed animals. The levels of these transporters were even lower than in the control LFD group. The decreased level of CD36 may lead to lower availability of FFA used for de novo lipid synthesis. FATP1 is acyl-CoA synthetase highly expressed in skeletal muscles, and it modulates fatty acid uptake as well as metabolism by converting fatty acids into fatty acyl-CoA [50]. In studies using FATP1-knockout mice, it has been shown that in the absence of this transporter, insulin-stimulated fatty acids uptake in skeletal muscle is significantly reduced [51]. In addition, FATP1 deletion was shown to protect mice from fat-induced insulin resistance and muscle lipid accumulation [51,52].

In our research, the level of FABPpm significantly increased in the HFD_+scrshRNA_ muscles and *Sptlc2* silencing resulted in an even greater, statistically significant increase compared with both control LFD and HFD_+scrshRNA_ muscles. Talanian et al. imply that the higher content of FABPpm in skeletal muscle results not so much in increased fatty acid influx but more enhanced their oxidation [53]. Additionally, an increase in levels of this protein during endurance training suggests that FABPpm expression in skeletal muscle may determine oxidative capacity [54].

Our research was based on *SPT* silencing in high-fat diet-fed animals. This enzyme is involved in the first steps of sphingolipid biosynthesis—condensation of serine and palmitoyl-CoA. The level of SPT activity may differ depending on the types of tissues and cells [55,56,57] or developmental stage of tissues [58]. Furthermore, the enzyme activity is affected by diet [59,60]. During our study we proved that the contents of mRNA for *Sptlc2* subunits and Sptlc2 protein were significantly increased in HFD_+scrshRNA_ muscles. *Sptlc2* silencing was clearly observed at both the mRNA and protein levels. The content of Sptlc2 protein dropped to values lower than those observed in the control LFD group. SPT is suggested to be a pivotal enzyme for the regulation of sphingolipid levels in cells. Regulation of sphingolipid synthesis at the SPT stage prevents the harmful accumulation of both intermediates (sphingolipid bases) and ceramide, whereas inhibiting sphingolipid synthesis at a later stage may result in the accumulation of intermediates, including sphinganine. [12]. In our research, *SPT* silencing led to the reduction of the content of ceramide intermediates, including sphinganine as well as sphingolipids which are products of ceramide catabolism, sphingosine and sphingosine-1-phosphate (S1P). A decrease in the amount of sphinganine and sphingosine was also observed in a study on cardiomyocyte-specific Sptlc2-deficient mice [61]. The use of myriocin as an SPT inhibitor also decreased the level of these compounds [62,63]. Of particular importance is the reduced content of sphingosine, which inhibits PKC and thus affects the glucose metabolism in skeletal muscle cells [64,65,66]. Elevated S1P levels in our research in the HFD_+scrshRNA_ group could be recognized as one of the important features of obesity [67,68]. It has been demonstrated, that saturated fatty acids induce an increase in S1P concentration [69]. In our study, we noted an increased level of S1P in HFD_+scrshRNA_ muscles and a decrease in the content of this compound in HFD_Sptlc2-shRNA_ muscles, which was most likely related to a decrease in SPH levels [70].

Regarding ceramide, the observed differences in the amount of this lipid were significant in all the three examined muscles. High-fat diet consumption contributed to an over-70% increase in ceramide content in the gastrocnemius muscle. By *Sptlc2* silencing, the impact of HFD was eliminated and the ceramide level was similar to those observed in LFD-fed animals. Moreover, in other studies, SPT inhibition had a significant effect on reducing ceramide accumulation. Studies on L6 cell lines have shown that inhibition of *SPT* expression in myocytes with a specific shRNA or cells incubated with myriocin, an SPT inhibitor, leads to a reduction of ceramide accumulation, which positively affects the activity of the insulin pathway [62,71]. Moreover, in mice with cardiomyocyte-specific deficiency of Sptlc2, an approximately 35% reduction in de novo ceramide synthesis was observed [61]. The results of studies conducted in animals fed HFD and treated with myriocin, also showed a decrease in muscle ceramide levels compared with animals fed HFD without administration of this inhibitor, which was associated with an improvement in insulin sensitivity despite the observed elevated muscle DAG levels in these animals [63]. In our present research, although in the HFD_Sptlc2-shRNA_ muscle we observed a significant decrease of ceramide level, it did not affect the other total lipid content. In both muscles, HFD_+scrshRNA_ and HFD_Sptlc2-shRNA_, we obtained similar, increased levels of total DAG, TAG, LCA-CoA and LCA-carnitine compared to the levels of these compounds in the muscles of LFD-fed animals. According to some studies which confirmed that it is impossible to utilize palmitoyl-CoA for ceramide synthesis via the SPT pathway, palmitate may be channeled into other pathways, such as those responsible for DAG synthesis [62,71]. In our study, *Sptlc2* gene silencing was local, which might be the reason for not obtaining similar results. However, interesting changes occurred in the content of DAG C16:0/16:0. Silencing of *Sptlc2* in the gastrocnemius muscle significantly increased the concentration of this lipid in high-fat diet-fed mice. Similar results were obtained by Ruangsiriluk et al. [72]. Although the total level of each lipid class was not altered, there were considerable changes at the subspecies level. These authors showed that the most frequently changed lipids in cells with inhibited ceramide synthesis were those containing palmitate. The lack of changes in total lipid content other than ceramides in the silenced muscles may also be associated with an increased rate of fatty acid oxidation. In our research, in the HFD_Sptlc2-shRNA_ muscles we observed increased levels of CPT1B1, which controls the entry of long-chain acyl CoA into mitochondria and thus facilitates the oxidation of fatty acids [73]. Bruce et al. showed that 20% upregulation of CPT1B in the muscles of rats fed HFD may decrease the triacylglycerol content and membrane-to-cytosolic ratio of diacylglycerol [72]. These authors also found that overexpression of CPT1 reduced IRS1 serine phosphorylation. In addition, numerous studies have shown that changes in the influx of fatty acids into the mitochondria are crucial in generating muscle insulin resistance [73,74,75,76].

Our results confirmed that HFD is the cause of disturbances related to the inulin signaling pathway and, consequently, to glucose metabolism. Under physiological conditions, the tyrosine kinase activity of insulin receptors is stimulated in response to insulin, leading to autophosphorylation and subsequent phosphorylation of tyrosine residues of insulin receptor substrate (IRS) [77].

In the work presented here, we observed inhibition of tyrosine phosphorylation (Tyr632) and upregulation of IRS1 serine phosphorylation (Ser1101) in HFD_+scrshRNA_ muscles. Inhibition of de novo ceramide synthesis has a positive effect on muscle sensitivity to insulin. In HFD_Sptlc2-shRNA_ muscles, the degree of phosphorylation of IRS tyrosine residues increased and that of serine residues decreased, which indicates an inhibition of the insulin pathway. Unfortunately, the mechanisms by which ceramide affected this protein remain unknown. Higher phosphorylation of tyrosine residues in IRS due to *SPT* silencing could have had an impact on PI3K, the next protein of the insulin signaling pathway. We found a significant decrease in the content of this protein in the HFD_+scrshRNA_ muscles. In HFD_Sptlc2-shRNA_ muscles the content of PI3K rose to a level much higher than that observed in the control LFD mice. This confirms that silencing the *Sptlc2* gene and decreasing ceramide production enhances the action of the insulin pathway also at this stage.

A key enzyme in the insulin pathway inhibited by ceramide is PKB/Akt. Experimental studies revealed that ceramides might weaken insulin activity through maintaining PKB/Akt in an inactive dephosphorylated state in two ways: firstly, by activation of atypical PKC isoforms (*ζ*/*λ*), which favors their combination with PKB/Akt and prevents the activation of PKB/Akt in response to insulin [78,79,80]; and secondly, ceramides indirectly suppress the PKB/Akt activity by stimulation of serine/threonine phosphatase PP2A [64]. In our research, we demonstrated that significant dephosphorylation of serine 473 in PKB/Akt occurs in high-fat diet-fed animals. The reduced ceramide content achieved by *SPT* gene silencing returned the degree of serine 473 phosphorylation to the control level, which is necessary for proper functioning of the insulin pathway. Other studies [62,63,71] also confirmed that ceramide synthesis inhibition by blocking SPT action increases the phosphorylation of PKB/Akt.

The stimulatory effect of insulin on IRS, PI3K and PKB/Akt is crucial for the subsequent translocation of GLUT4 to the plasma membrane. The observed growth in the quantity or phosphorylation level of these proteins in HFD_Sptlc2-shRNA_ muscles is reflected in the increase of GLUT4 content. All the changes observed in our study, resulting from the silencing of *SPT* expression, lead to the improvement of the insulin pathway.

## 5. Conclusions

Overall, the results of our research indicate that the consumption of HFD leads to an increase in plasma free fatty acids concentration and an increase in the level of lipids, including ceramides, DAG and LCACoA in skeletal muscle. The observed increase in lipid content was accompanied by inhibition of the activity of insulin pathway proteins, as evidenced by a decrease in the level and/or degree of their phosphorylation. The results obtained demonstrate that *Sptlc2* gene silencing decreases the accumulation of ceramides without affecting the content of other lipids and results in an improvement in the sensitivity of skeletal muscles to insulin. The results of these studies suggest that inhibition of ceramide synthesis could be considered as a potential therapeutic target for lipid-induced insulin resistance.

## Figures and Tables

**Table 1:** Impact of High-Fat Diet Consumption on Plasma Free-Fatty Acid Concentration.

	C14:0	C16:0	C18:0	C18:1	C18:2	C20:0
Control LFD	7.62 (5.52–8.44)	69.44 (67.15–78.82)	29.69 (27.58–32.64)	67.71 (63.30–75.59)	58.45 (51.44–71.57)	1.18 (0.90–1.29)
HFD	17.22 (16.46–17.51) *	96.74 (83.98–102.50) *	61.43 (58.91–64.31) *	143.9 (138.1–147.8) *	137.40 (131.90–143.50) *	3.99 (3.67–4.29) *
	**C20:4**	**C22:0**	**C24:0**	**C24:1**	**Total**	
Control LFD	9.41 (8.79–10.87)	3.90 (3.58–4.22)	3.71 (3.32–4.72)	0.17 (0.14–0.18)	266.30 (253.80–280.80)	
HFD	19.71 (19.21–20.42) *	11.01 (10.79–11.73) *	8.34 (7.51–8.73) *	0.34 (0.31–0.42) *	509.70 (486.50–517.10) *	

Values are medians (interquartile range); *n* = 8 per group. Significance by Mann–Whitney U test, *: *p* < 0.05 vs. control LFD.

**Table 2:** The Impact of *Sptlc2* Silencing on the Content of Ceramide in the Mouse Gastrocnemius Muscle.

	C14:0-Cer	C16:0-Cer	C18:0-Cer	C18:1-Cer
Control LFD	0.029 (0.028–0.031)	1.93 (1.84–2.13)	16.82 (15.36–18.20)	0.55 (0.49–0.63)
HFD_+scrshRNA_	0.034 (0.027–0.038)	3.30 (2.85–3.48) *	29.91 (28.12–33.03) *	0.67 (0.61–0.71) *
HFD_Sptlc2-shRNA_	0.024 (0.023–0.025) *^/#^	1.82 (1.67–1.89) ^#^	18.63 (17.18–20.37) ^#^	0.28 (0.25–0.29) *^/#^
	**C20:0-Cer**	**C22:0-Cer**	**C24:0-Cer**	**C24:1-Cer**
Control LFD	0.27 (0.25–0.30)	0.75 (0.71–0.85)	1.24 (1.16–1.36)	3.78 (3.49–4.22)
HFD_+scrshRNA_	0.57 (0.47–0.65) *	1.44 (1.29–1.60) *	2.57 (2.38–2.65) *	5.06 (4.53–5.39) *
HFD_Sptlc2-shRNA_	0.29 (0.28–0.33) ^#^	0.83 (0.81–0.88) ^#^	1.49 (1.39–1.57) *^/#^	2.61 (2.51–2.87) *^/#^

Values are medians (interquartile range); *n* = 8 per group. Significance by Mann–Whitney U test, *: *p* < 0.05 vs. control LFD, #: *p* < 0.05 vs. HFD_+scrshRNA_.

**Table 3:** The Impact of *Sptlc2* Silencing on the Content of Sphingosine, Sphinganine and Sphingosine-1-Phosphaate in the Mouse Gastrocnemius Muscle.

	Sphingosine	Sphinganine	Sphingosine-1-Phosphate
Control LFD	0.38 (0.36–0.21)	0.20 (0.19–0.21)	0.035 (0.033–0.045)
HFD_+scrshRNA_	0.52 (0.46–0.63) *	0.27 (0.24–0.32) *	0.057 (0.052–0.065) *
HFD_Sptlc2-shRNA_	0.41 (0.37–0.46) ^#^	0.18 (0.16–0.19) ^#^	0.040 (0.036–0.043) ^#^

Values are medians (interquartile range); *n* = 8 per group. Significance by Mann–Whitney U test, *: *p* < 0.05 vs. control LFD, #: *p* < 0.05 vs. HFD_+scrshRNA_.

**Table 4:** The Impact of *Sptlc2* Silencing on the Content of DAG in the Mouse Gastrocnemius Muscle.

	C16:0/16:0	C18:0/18:0	C18:1/18:1	C18:0/20:0	C16:0/18:0
Control LFD	13.10 (11.99–14.22)	2.06 (1.88–2.28)	20.27 (18.87–23.35)	3.30 (3.50–3.93)	83.55 (72.59–93.61)
HFD_+scrshRNA_	16.26 (16.75–18.29) *	2.89 (2.74–3.17) *	22.65 (21.39–25.05)	7.03 (6.58–8.05) *	138.70 (134.20–162.70) *
HFD_Sptlc2-shRNA_	19.15 (17.54–20.10) *^/#^	1.99 (1.54–2.37) ^#^	20.45 (18.79–24.42)	6.30 (5.71–6.96) *	143.10 (128.20–155.50) *
	**C18:0/18:1**	**C16:0/18:1**	**C16:0/18:2**	**C18:0/18:2**	
Control LFD	21.31 (19.68–24.36)	33.51 (28.28–36.33)	40.24 (33.12–42.59)	0.99 (0.80–1.23)	
HFD_+scrshRNA_	22.62 (21.11–26.33)	39.97 (37.53–42.99) *	65.92 (55.17–76.33) *	1.98 (1.76–2.18) *	
HFD_Sptlc2-shRNA_	26.91 (25.84–28.08) *^/#^	34.99 (32.96–38.00) ^#^	63.25 (59.44–68.45) *	2.42 (2.10–2.88) *^/#^	

Values are medians (interquartile range); *n* = 8 per group. Significance by Mann–Whitney U test, *: *p* < 0.05 vs. control LFD, #: *p* < 0.05 vs. HFD_+scrshRNA_.

**Table 5:** The Impact of *Sptlc2* Silencing on the Content of Acyl-CoA in the Mouse Gastrocnemius Muscle.

	C14:0-CoA	C16:0-CoA	C18:0-CoA	C18:1-CoA
Control LFD	0.116 (0.096–0.133)	0.802 (0.770–0.943)	0.719 (0.655–0.824)	2.55 (2.23–2.81)
HFD_+scrshRNA_	0.204 (0.172–0.223) *	0.922 (0.903–1.113) *	1.183 (1.067–1.336) *	2.74 (2.26–3.13)
HFD_Sptlc2-shRNA_	0.197 (0.184–2.226) *	1.113 (0.857–1.36) *	1.238 (1.167–1.390) *	2.66 (2.35–2.95)
	**C20:0-CoA**	**C22:0-CoA**	**C24:0-CoA**	**C24:1-CoA**
Control LFD	0.016 (0.015–0.017)	0.022 (0.019–0.023)	0.028 (0.024–0.031)	0.0152 (0.0134–0.0210)
HFD_+scrshRNA_	0.018 (0.016–0.022)	0.031 (0.029–0.036) *	0.031 (0.030–0.034)	0.0083 (0.0081–0.0099) *
HFD_Sptlc2-shRNA_	0.019 (0.018–0.022) *	0.036 (0.030–0.039) *	0.030 (0.029–0.034)	0.0086 (0.0079–0.0099) *

Values are medians (interquartile range); *n* = 8 per group. Significance by Mann–Whitney U test, *: *p* < 0.05 vs. control LFD.

**Table 6:** The Impact of *Sptlc2* Silencing on the Content of Acyl-Carnitine in the Mouse Gastrocnemius Muscle.

	C14:0 Carnitine	C16:0 Carnitine	C18:0 Carnitine	C18:1 Carnitine
Control LFD	1.46 (1.32–1.63)	2.41 (2.24–2.57)	0.74 (0.61–0.78)	7.00 (6.41–7.29)
HFD_+scrshRNA_	1.26 (1.03–1.33) *	2.94 (2.71–3.11) *	1.15 (1.13–1.40) *	7.45 (6.80–7.67)
HFD_Sptlc2-shRNA_	1.18 (1.10–1.34) *	2.74 (2.46–3.15) *	1.13 (1.05–1.28) *	7.44 (6.80–7.91)

Values are medians (interquartile range); *n* = 8 per group. Significance by Mann–Whitney U test, *: *p* < 0.05 vs. control LFD.

## Data Availability

Data are contained within the article or supplementary materials.

## Supplementary Materials

The following are available online at https://www.mdpi.com/article/10.3390/cells11071123/s1, Figure S1: Full unedited gel for Figure SPTLC, Figure S2: Full unedited gel for Figure pAkt(S473)/Akt, Figure S3: Full unedited gel for Figure CPT1B, Figure S4: Full unedited gel for Figure FABPpm, Figure S5: Full unedited gel for Figure GLUT4, Figure S6: Full unedited gel for Figure FATP1, Figure S7: Full unedited gel for Figure CD36, Figure S8: Full unedited gel for Figure PI3K, Figure S9: Full unedited gel for Figure pIRS-1(S1101)/IRS-1 and IRS1/pIRS-1(Y632).
