# Supplementary material for: Serine Palmitoyltransferase Gene Silencing Prevents Ceramide Accumulation and Insulin Resistance in Muscles in Mice Fed a High-Fat Diet
Source: Cells. 2022 Mar 26;11(7):1123. doi: 10.3390/cells11071123 (PMC8997855; doi:10.3390/cells11071123)
Supplement: Supplementary file 1 [file cells-11-01123-s001.zip › cells-1630712-supplementary.pdf]

Supplementary Figures

The sequence of application the samples into the gel wells was individually set for particular proteins.

Figure S1 Full unedited gel for Figure SPTLC. **The sequence of application the samples into the gel wells:** 1-4 lane –LFD sample, 5-8 lane – HFD$_{+Sptlc2}$ sample, 22-25 lane – HFD $_{Sptlc2\text{-}shRNA}$ sample, 26 lane – Protein standard, the rest of the lane – other samples (not analyzed).

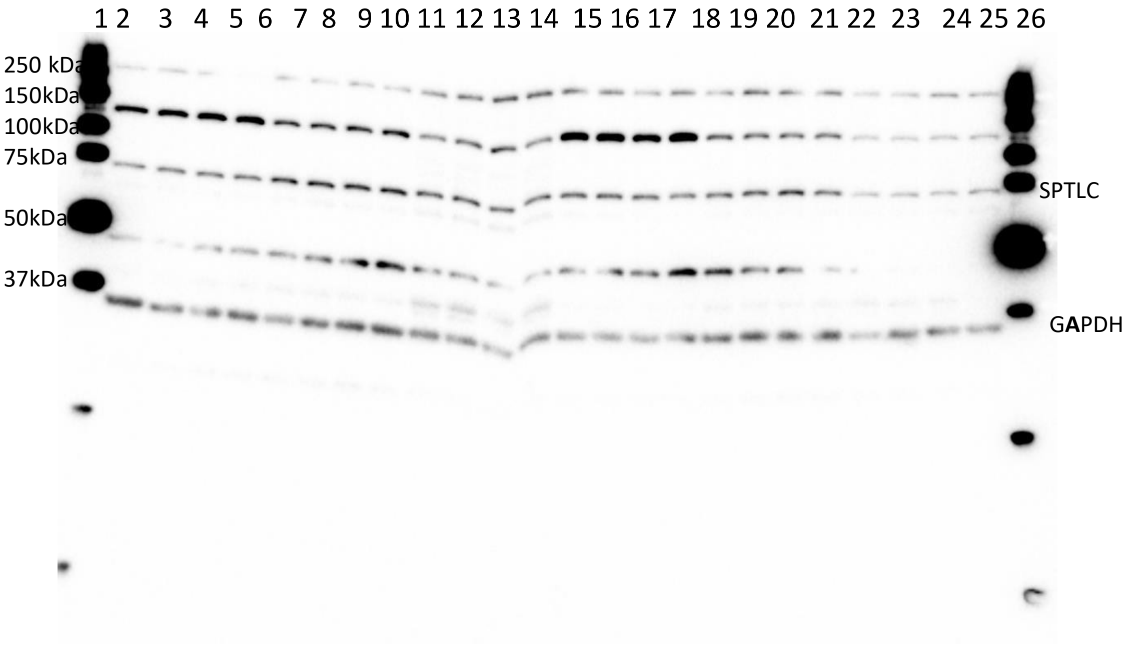

Figure S2 Full unedited gel for Figure pAkt(S473)/Akt. **The sequence of application the samples into the gel wells:** 1 lane – Protein standard, 2-5 lane – LFD sample, 6-9 lane – HFD$_{+Sptlc2}$ sample, 22-25 lane – HFD $_{Sptlc2-shRNA}$sample, 26 lane – Protein standard, the rest of the lane – other samples (not analyzed).

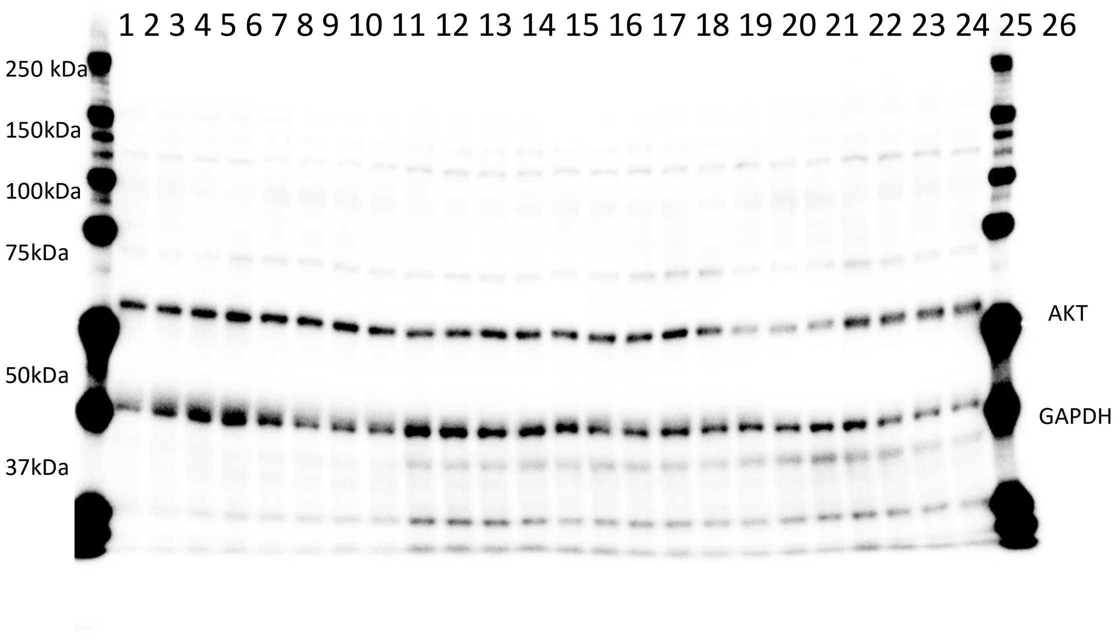

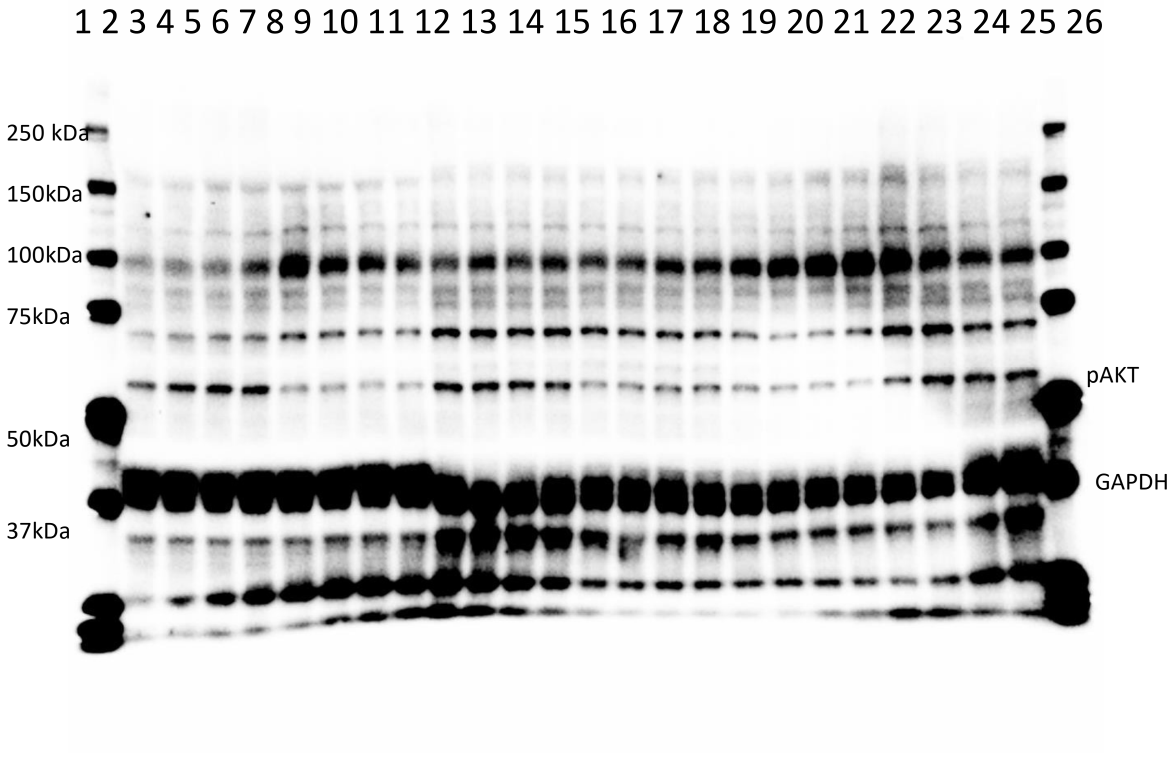

Figure S3 Full unedited gel for Figure CPT1B. **The sequence of application the samples into the gel wells:** 1 lane – Protein standard, 2-5 lane – LFD sample, 6-9 lane – $HFD_{+Sptlc2}$ sample, 18-21 lane – $HFD_{Sptlc2\text{-}shRNA}$ sample,26 lane – Protein standard, the rest of the lane – other samples (not analyzed).

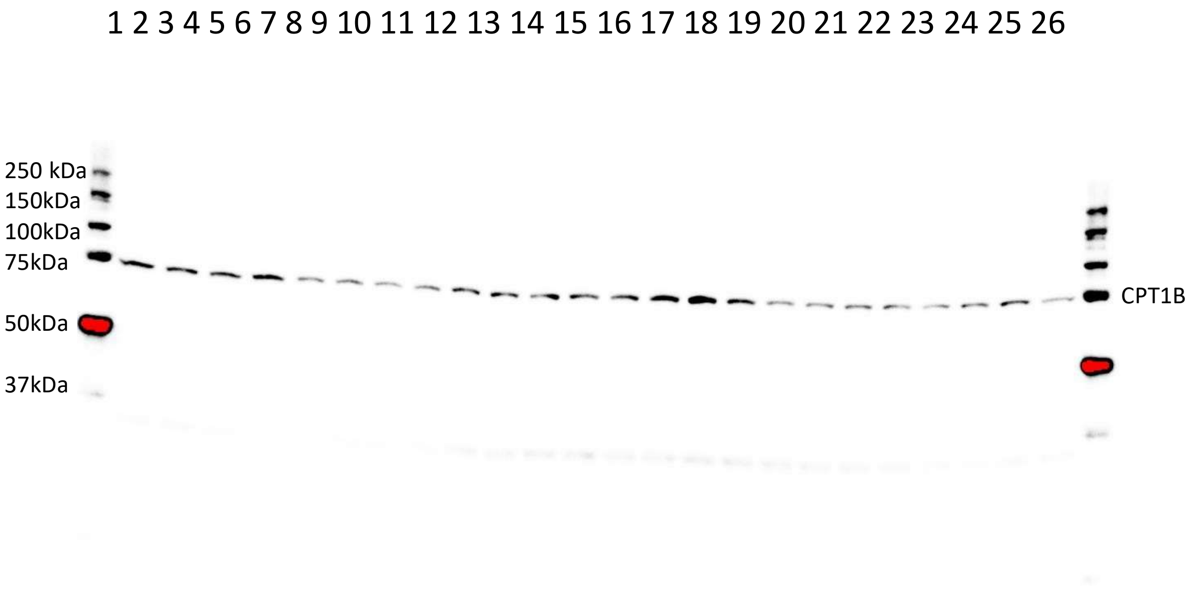

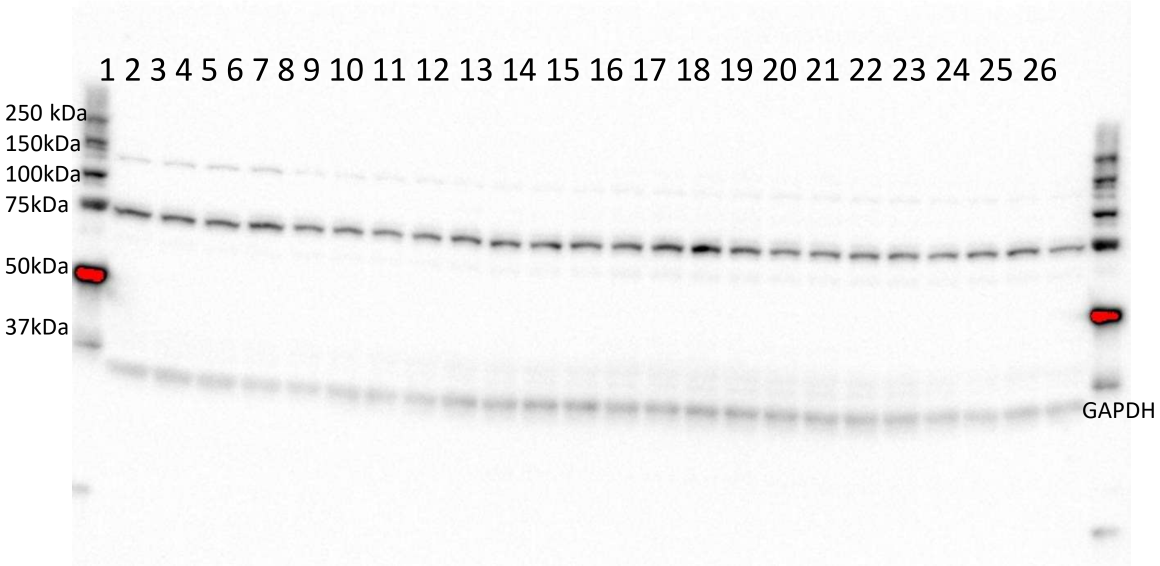

Figure S4 Full unedited gel for Figure FABPpm. **The sequence of application the samples into the gel wells:** 1 lane – Protein standard, 2-5 lane – LFD sample, 6-9 lane – HFD$_{+Sptlc2}$ sample, 18-21 lane – HFD $_{Sptlc2-shRNA}$ sample, the rest of the lane – other samples (not analyzed).

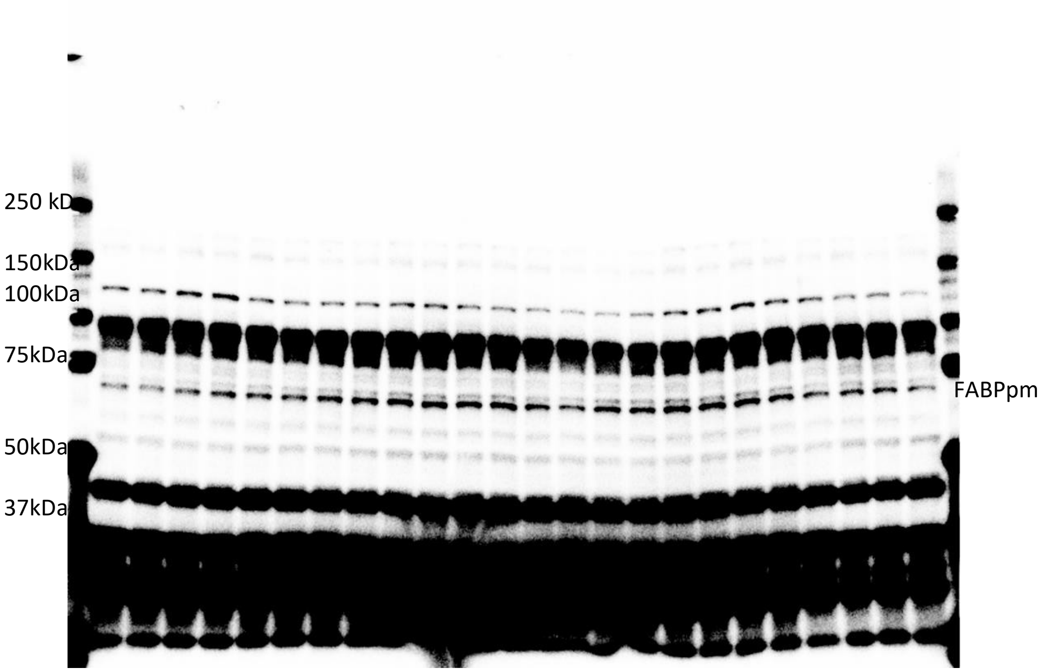

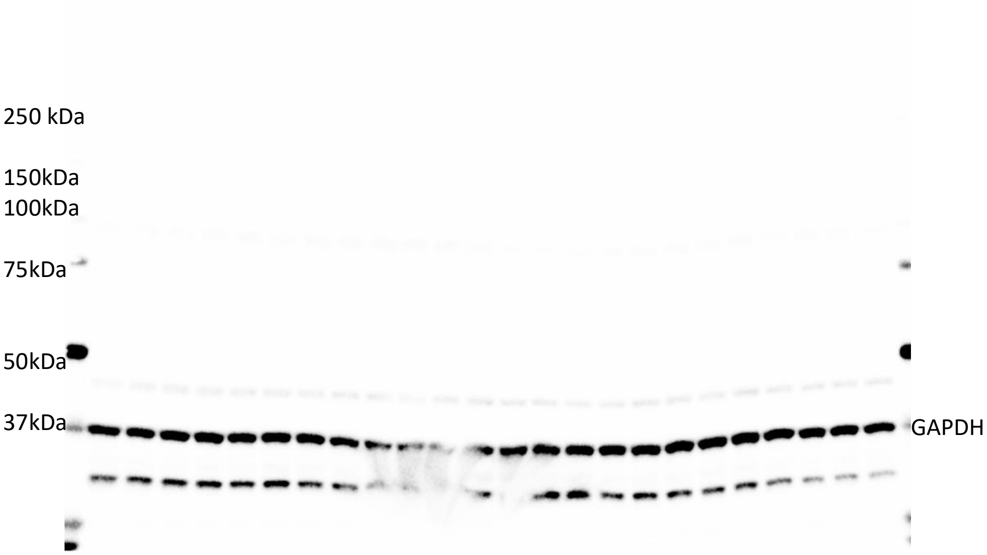

Figure S5 Full unedited gel for Figure GLUT4. **The sequence of application the samples into the gel wells:** 1 lane – Protein standard, 2-5 lane – LFD sample, 6-9 lane – HFD$_{+Sptlc2}$ sample, 18-21 lane – HFD $_{Sptlc2-shRNA}$ sample, 26 lane – Protein standard, the rest of the lane – other samples (not analyzed).

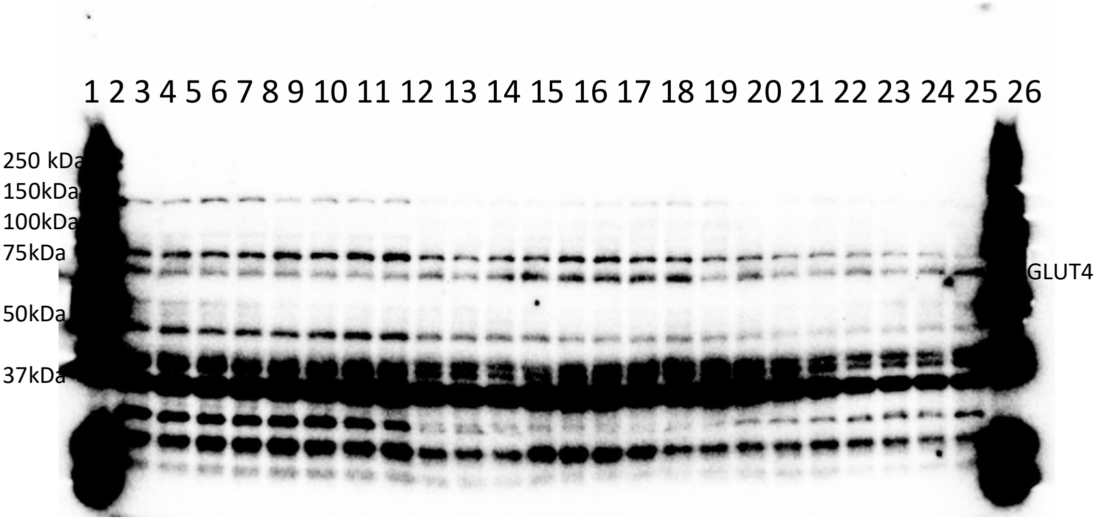

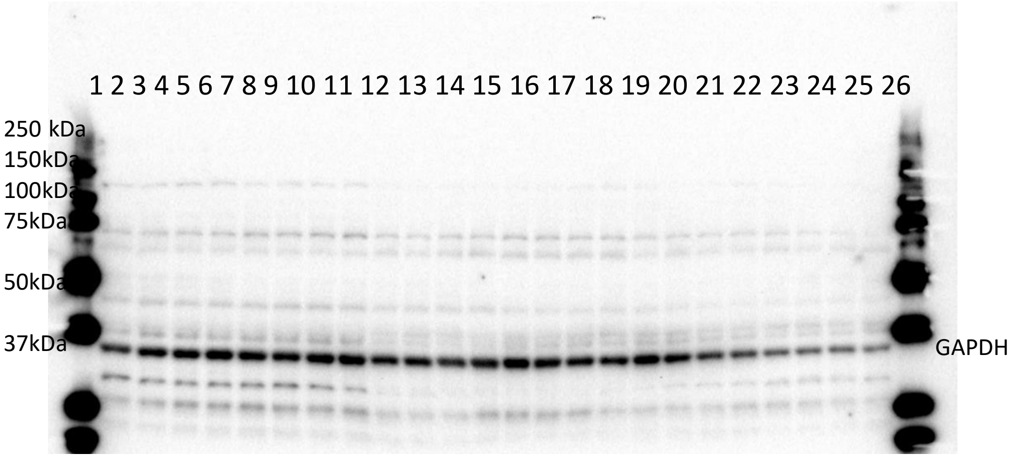

Figure S6 Full unedited gel for Figure FATP1. **The sequence of application the samples into the gel wells:** 1 lane – Protein standard, 2-5 lane – LFD sample, 6-9 lane – $HFD_{+Sptlc2}$ sample, 18-21 lane – HFD $_{Sptlc2\text{-}shRNA}$ sample, 26 lane – Protein standard, the rest of the lane – other samples (not analyzed).

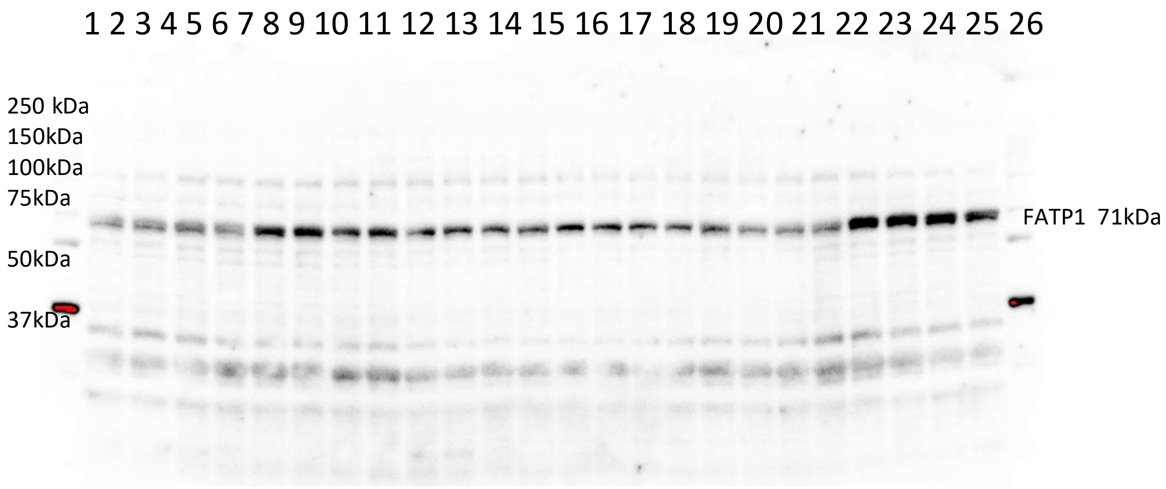

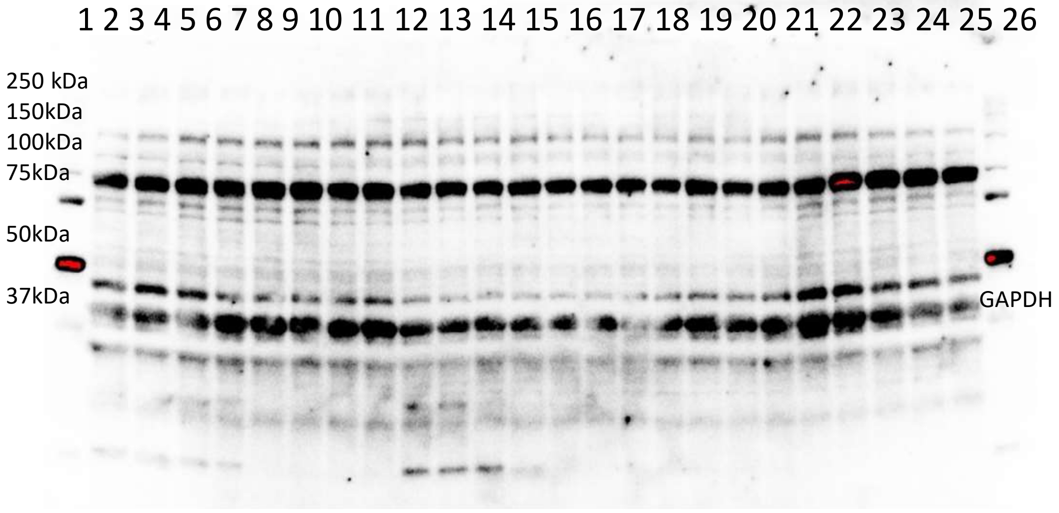

Figure S7 Full unedited gel for Figure CD36. **The sequence of application the samples into the gel wells:** 1 lane – Protein standard, 2-5 lane – HFD$_{+Sptlc2}$ sample, 6-9 lane – LFD sample, 18-21 lane – HFD $_{Sptlc2\text{-}shRNA}$ sample, 26 lane – Protein standard, the rest of the lane – other samples (not analyzed).

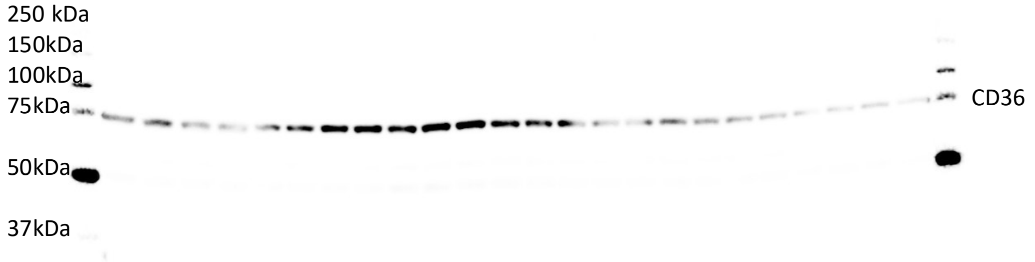

Figure S8 Full unedited gel for Figure PI3K. **The sequence of application the samples into the gel wells:** 1 lane – Protein standard, 2-5 lane – LFD sample, 6-9 lane – HFD$_{+Sptlc2}$ sample, 18-21 lane – HFD $_{Sptlc2\text{-}shRNA}$ sample, 26 lane – Protein standard, the rest of the lane – other samples (not analyzed).

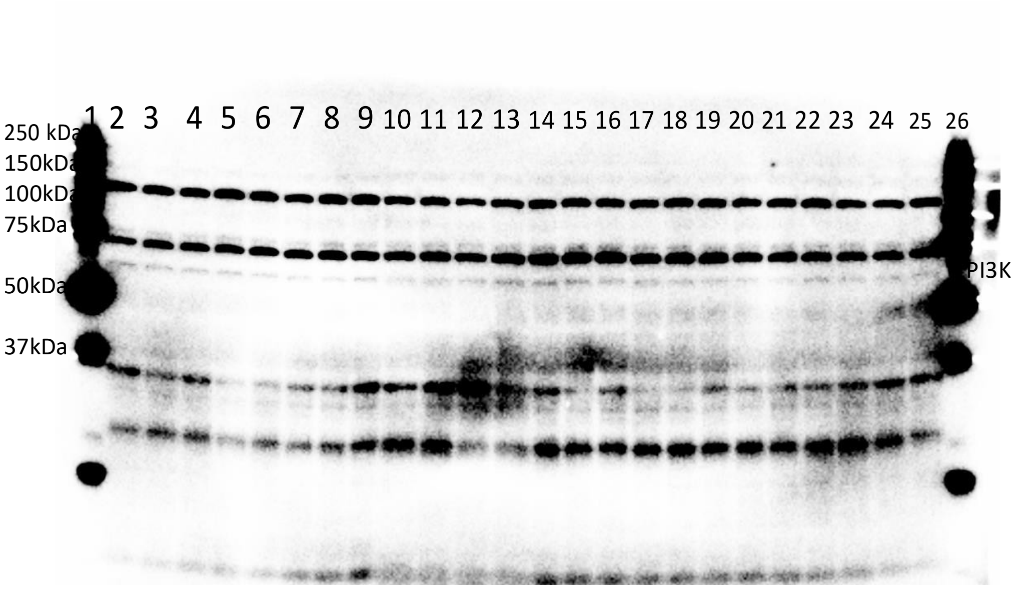

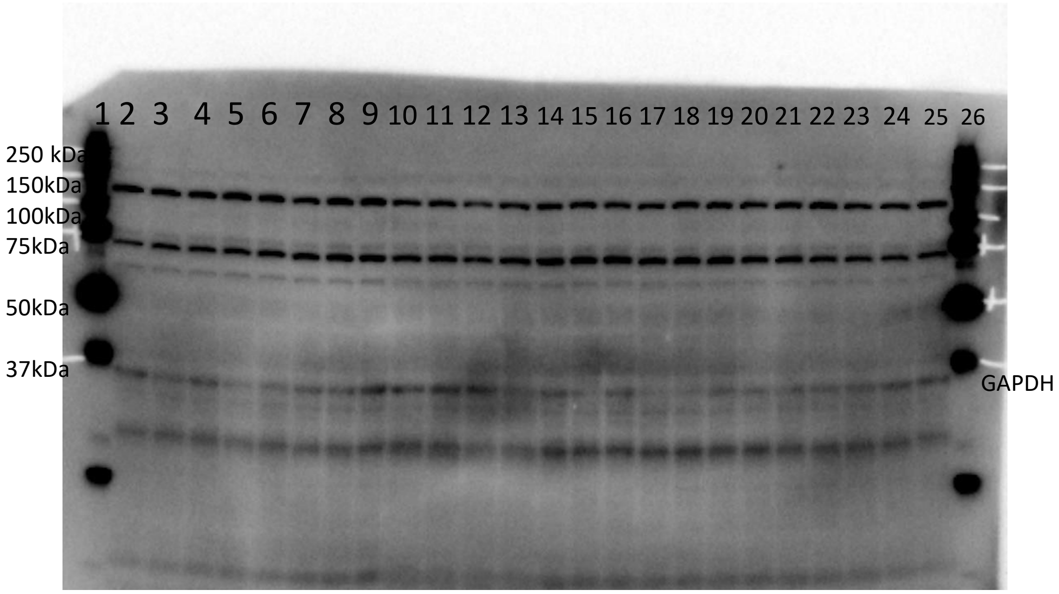

Figure S9 Full unedited gel for Figure pIRS-1(S1101)/IRS-1 and IRS1/pIRS-1(Y632) . **The sequence of application the samples into the gel wells:** 1 lane – Protein standard, 2-5 lane – LFD sample, 6-9 lane – HFD$_{+Sptlc2}$ sample, 22-25 lane – HFD $_{Sptlc2-shRNA}$ sample, 26 lane – Protein standard, the rest of the lane – other samples (not analyzed).

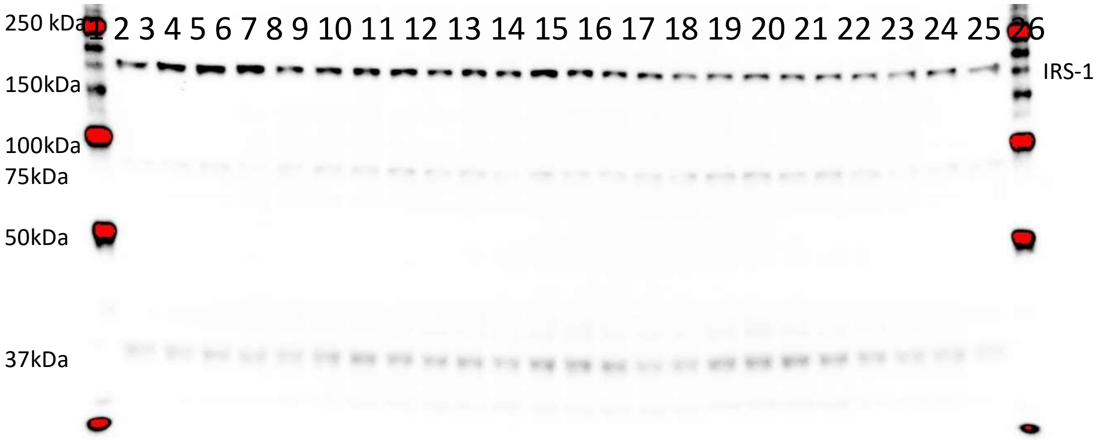

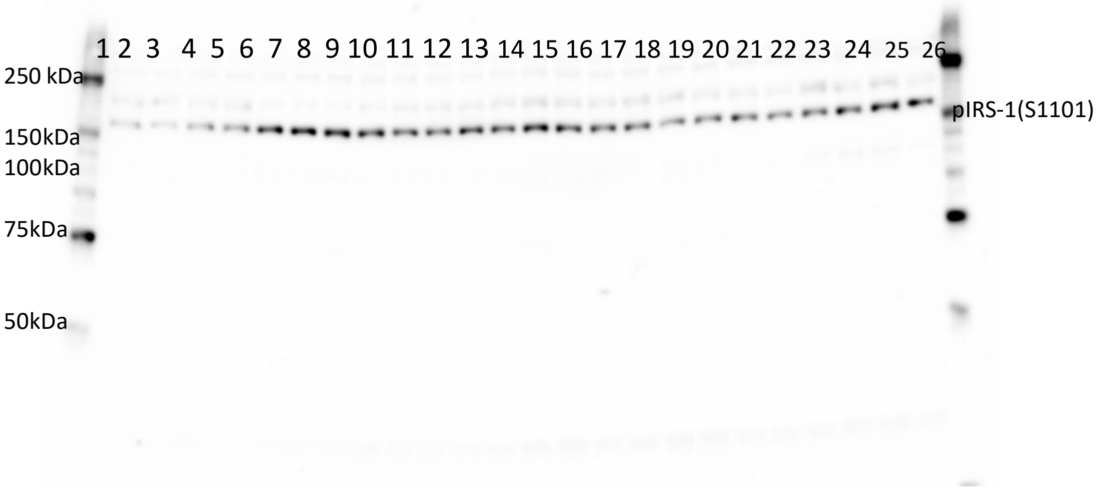
1 2 3 4 5 6 7 8 9 10 11 12 13 14 15 16 17 18 19 20 21 22 23 24 25 26
250 kDa
150kDa
100kDa
75kDa
50kDa
pIRS-1(S1101)

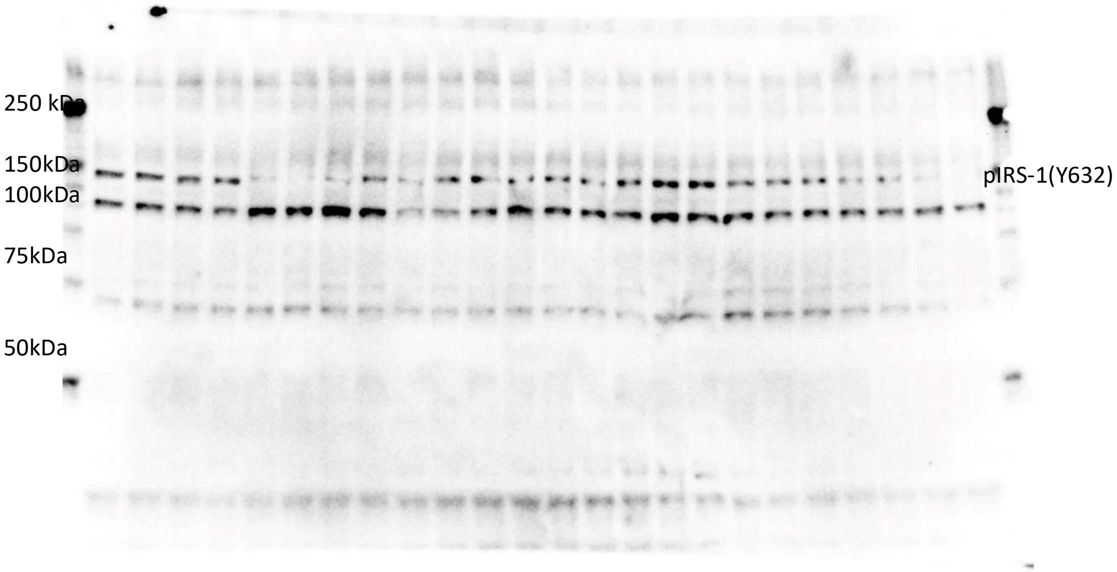
250 kDa
150kDa
100kDa
75kDa
50kDa
pIRS-1(Y632)

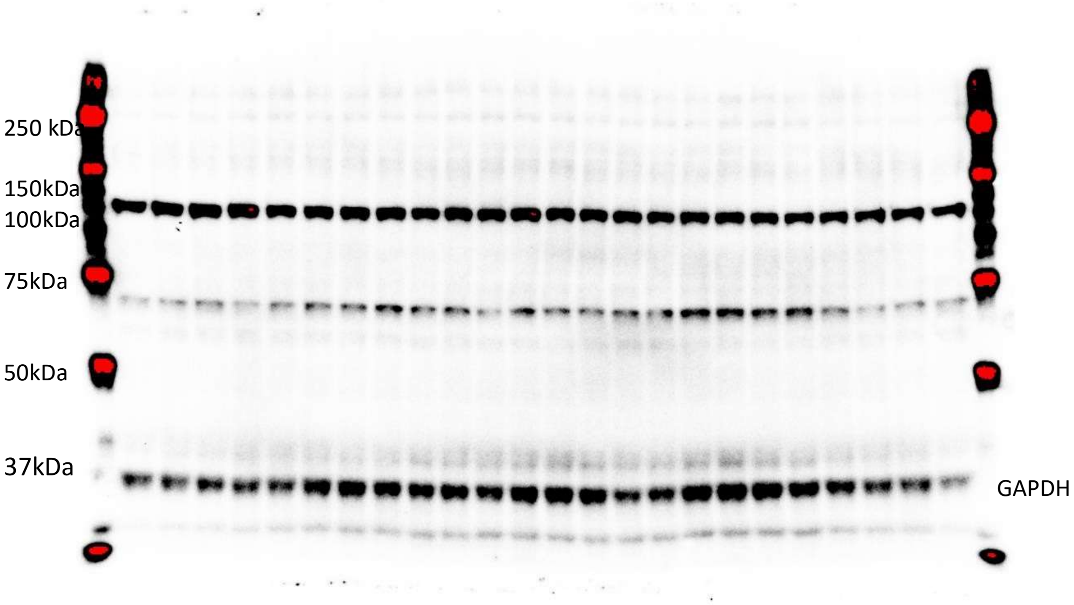
250 kDa
150kDa
100kDa
75kDa
50kDa
37kDa
GAPDH
